# Impact of Heavy Metal Pollution in the Environment on the Metabolic Profile of Medicinal Plants and Their Therapeutic Potential

**DOI:** 10.3390/plants13060913

**Published:** 2024-03-21

**Authors:** Dana-Mihaela Asiminicesei, Daniela Ionela Fertu, Maria Gavrilescu

**Affiliations:** 1 Department of Environmental Engineering and Management, “Cristofor Simionescu” Faculty of Chemical Engineering and Environmental Protection, “Gheorghe Asachi” Technical University of Iasi, 73 Prof. D. Mangeron Blvd., 700050 Iasi, Romania; dana-mihaela.asiminicesei@student.tuiasi.ro; 2 Department of Pharmaceutical Sciences, Faculty of Medicine and Pharmacy, “Dunarea de Jos” University of Galati, 35 Al. I. Cuza Street, 800002 Galati, Romania; 3 Academy of Romanian Scientists, 3 Ilfov Street, 050044 Bucharest, Romania

**Keywords:** antioxidant defense mechanisms, bioactive compound synthesis, environmental factors, health risks, epigenetic modifications, oxidative stress, redox equilibrium, regulatory challenges, root-shoot signaling

## Abstract

The paper provides a comprehensive examination of heavy metal stress on medicinal plants, focusing on its impact on antioxidant capacity and biosynthetic pathways critical to their therapeutic potential. It explores the complex relationship between heavy metals and the physiological and biochemical responses of medicinal plants, highlighting how metal stress disrupts biosynthetic pathways, altering concentrations of secondary metabolites. This disruption may compromise the overall quality and efficacy of medicinal plants, requiring a holistic understanding of its cumulative impacts. Furthermore, the study discusses the potential of targeted genetic editing to enhance plant resilience against heavy metal stress by manipulating genes associated with antioxidant defenses. This approach represents a promising frontier in safeguarding medicinal plants in metal-contaminated environments. Additionally, the research investigates the role of phytohormone signaling in plant adaptive mechanisms to heavy metal stress, revealing its influence on biochemical and physiological responses, thereby adding complexity to plant adaptation. The study underscores the importance of innovative technologies and global cooperation in protecting medicinal plants’ therapeutic potential and highlights the need for mitigation strategies to address heavy metal contamination effectively.

## 1. Introduction

### 1.1. Importance of Medicinal Plants and Their Compounds

Medicinal plants (herbs) play a central role in promoting human health and sustainable healthcare practices due to their rich array of bioactive compounds. Rooted in tradition and wisdom, these botanical resources offer a nature-driven and holistic approach to improving human life. The diverse range of bioactive compounds found within medicinal plants contributes to their significance in supporting well-being [1]. Alkaloids, flavonoids, terpenoids, and polyphenols stand as chemicals of therapeutic effects. Their profound significance lies not only in the richness of diversity they bring, but also in the versatile roles they play—from antioxidant defenders shielding cells against oxidative stress to anti-inflammatory agents soothing the body’s unrestrained responses [2,3,4] In addition to their antioxidant properties, many medicinal plants also possess anticarcinogenic properties, meaning they can inhibit the growth and spread of cancer cells [5,6].

However, medicinal plants are more than just their medical properties. They hold onto the cultural past and traditional ways of healing, woven deeply into the social and cultural traditions of communities worldwide. For generations, indigenous societies have depended on the knowledge passed down to them to use these plants to treat various health issues. While some of the claims about these plants have been questioned and improved over time, the practice of using plant-based treatments is rooted in centuries of real-life experience and observation. The historical roots of medicinal plant usage can be traced back to ancient civilizations, with records dating as far back as Mesopotamia circa 2600 BC, where substances like Cedar oil, Cypress, Licorice, Myrrh, and Poppy juice were documented for their medicinal properties [1]. Similarly, Ancient Egyptian medical texts reveal the utilization of plants such as bishop’s weeds (*Ammi majus*) in treating conditions like vitiligo, a disorder characterized by pigment loss. Remarkably, the therapeutic legacy of these plants endures to this day, with modern pharmacology harnessing their active compounds for the management of conditions like psoriasis and T-cell lymphoma [7]. Merged into the historical chain of societies, these plants have been reliable companions in the pursuit of health and recovery. Passed down through generations, indigenous knowledge openly connects with the leaves, roots, and blossoms of these plants, serving as a living testament to the enduring relationship between humanity and the natural world [8].

In the perspective of modern healthcare, medicinal plants serve as fertile grounds for drug discovery and development. From the heart of the rainforest to the meadows, these botanical reservoirs inspire scientists to unveil novel compounds that may become the cornerstones of future pharmaceuticals [9,10]. Moreover, the synergy between traditional wisdom and modern science underscores a harmonious relationship that underpins efforts to improve health outcomes. This symbiotic relationship resonates with the global imperative for sustainability, as reflected in the eco-conscious practices of cultivating and utilizing medicinal plants. By embracing natural remedies while prioritizing environmental stewardship, healthcare practitioners and policymakers pave the way for a greener and more sustainable healthcare paradigm [5]. In the broader context of environmental conservation, medicinal plants emerge not only as healers but also as custodians of ecosystems, playing a vital role in preserving biodiversity and ecological equilibrium [11,12]. Their conservation transcends mere botanical concern; it represents a collective commitment to safeguarding the complex web of life that sustains humanity and the natural world alike.

This paper is dedicated to investigating the impact of environmental pollutants on medicinal plant-derived active compounds with antioxidant properties. The research focuses on understanding how pollutants as heavy metals affect the therapeutic properties of the active compounds in medicinal plants, aiming to provide insights into the potential consequences of pollution and strategies for mitigating these effects.

### 1.2. Medicinal Plants and Their Bioactive Compounds

Medicinal plants are essential for human health and sustainable healthcare due to their diverse bioactive compounds. These include alkaloids, flavonoids, terpenoids, and polyphenols, known for their antioxidant, anti-inflammatory, antimicrobial, and anticancer properties [13,14]. They remain a significant resource for traditional medicine, particularly in regions with limited access to modern healthcare. Many pharmaceutical drugs have originated from these plants, inspiring the development of new medications [15,16]. Their cultivation and use are more environmentally friendly than synthetic pharmaceuticals, aligning with sustainable healthcare practices [17,18]. These plants contribute to biodiversity conservation and ecosystem balance. Moreover, they play an essential role in complementary medicine, offering holistic approaches to well-being [19]. Their compounds are increasingly used in nutraceuticals and functional foods for health enhancement and disease prevention. They also provide supportive care for chronic diseases alongside conventional treatments, thanks to their antioxidant and anti-inflammatory properties [19].

Medicinal plants allow personalized healthcare considering genetic, lifestyle, and environmental factors. They support livelihoods, particularly in rural areas where they are grown, bridging cultural, scientific, ecological, and economic dimensions [20,21]. Advancements in nanotechnology, pharmacogenomics, and phytopharmaceutical formulations offer promising avenues for enhancing their therapeutic potential [22,23]. Recognizing and capitalizing these natural resources are essential for advancing healthcare, sustainability, and preserving cultural knowledge [20,21].

## 2. Medicinal Plant Compounds with Antioxidant Activity

### 2.1. Key Medicinal Plant Compounds Known for Their Antioxidant Properties

Understanding the diverse classes of medicinal plant compounds and their antioxidant activities is essential for harnessing their therapeutic potential. Research into the mechanisms of action and synergistic effects of these compounds can provide valuable insights for the development of novel antioxidant-rich interventions in medicine and nutrition.

Antioxidants can be classified based on their origin, activity, and suitability for the efficient synthesis of nanoparticles. The key medicinal plant compounds known for their antioxidant properties are categorized as follows [6,24,25,26]:

- Polyphenols: represent a group of naturally occurring compounds characterized by the presence of multiple phenol structural units. They are widely distributed in plants and have gained significant attention for their antioxidant properties [4]. Flavonoids, phenolic acids, lignans, and stilbenes are subcategories of polyphenols found in various medicinal plants. Quercetin, resveratrol, and catechins are well-known polyphenols with potent antioxidant activities [27,28].
- Flavonoids: constitute a major subclass of polyphenols and are renowned for their antioxidant, anti-inflammatory, and anticancer properties. They are abundant in fruits, vegetables, and medicinal herbs. Quercetin (found in apples, onions), epicatechin (abundant in green tea, cocoa), and hesperidin (citrus fruits) are prominent flavonoids with notable antioxidant effects [29,30].
- Alkaloids: e a diverse group of nitrogen-containing compounds with varying pharmacological activities. Many alkaloids derived from medicinal plants exhibit antioxidant potential, contributing to their therapeutic effects [2]. Berberine (found in *Berberis* species), caffeine (present in coffee beans), morphine (from *Papaver somniferum*) are examples of alkaloids known for their antioxidant properties [31,32].
- Carotenoids: are pigments responsible for the red, orange, and yellow colors in various fruits and vegetables. They serve as antioxidants and are vital for human health, especially in protectting against oxidative stress. Beta-carotene (abundant in carrots, sweet potatoes), lutein (found in spinach, kale), and lycopene (present in tomatoes) are well-studied carotenoids with antioxidant benefits [33,34].
- Terpenoids (terpenes): are a large and diverse class of compounds derived from the isoprene unit. They are found in essential oils and resinous exudates of medicinal plants, contributing to their antioxidant and anti-inflammatory activities [35]. Limonene (citrus fruits), menthol (mint), and curcumin (turmeric) are terpenoids known for their antioxidant and medicinal properties.
- Phenolic acids: are a subgroup of polyphenols with a phenolic ring and an acidic moiety. They are abundant in fruits, vegetables, and whole grains, playing a crucial role in the antioxidant defense system [36,37]. Ferulic acid (present in whole grains), caffeic acid (found in coffee), and rosmarinic acid (in rosemary and sage) are phenolic acids with antioxidant potential.
- Saponins: are glycosides with a hydrophobic aglycone, exhibiting diverse biological activities, including antioxidant effects. They are commonly found in various medicinal plants [38]. Examples of saponins with antioxidant properties are ginsenosides (from *Panax ginseng*), quillaja saponins (from *Quillaja saponaria*), and soyasaponins (from soybeans).

Antioxidants can be found in various natural extracts prepared from different available species such as plants, fungi, bacteria, algae, lichens, and actinomycetes. These extracts rich in antioxidants can act as both reducing and stabilizing agents, making them useful for creating metallic nanoparticles through a process known as green synthesis. This method surpasses classical methods as it is low-cost, environmentally friendly, and does not require high pressure, energy, temperature, or external toxic chemicals [25,39].

The antioxidant activity of these extracts is still a challenge for analytical chemistry as there is no universal test for measuring total antioxidant capacity (TAC) [40,41,42]. However, there are many in vitro chemical tests that quantify the antioxidant scavenging activity of free radicals and their ability to chelate metals and reduce free radical damage. These tests provide significant insights into the mechanisms by which substances combat oxidative stress and free radical damage, which are implicated in various diseases and aging processes.

### 2.2. Significance of Antioxidant Activity in Sustaining Plant Health

The significance of antioxidant activity in maintaining plant health is multifaceted and decisive for the overall well-being of plants. Antioxidants play a key role in protecting plants from various environmental stressors and physiological processes. Antioxidant activity is a cornerstone of plant health, safeguarding against environmental challenges, supporting vital physiological processes, and contributing to the overall resilience and longevity of plants in their ecosystems. Understanding and harnessing the mechanisms of antioxidant defense in plants are essential for improving crop yield, promoting sustainable agriculture, and preserving biodiversity [6,43].

Antioxidants help plants combat oxidative stress, which arises due to an imbalance between the production of reactive oxygen species (ROS) and the plant’s ability to detoxify them. ROS are generated during normal metabolic processes and are exacerbated by environmental stressors such as UV radiation, pollutants, and pathogen attacks [44,45]. Therefore, antioxidants contribute to the plant’s defense mechanisms against pathogens. When a plant encounters pathogenic microorganisms, it often triggers an oxidative burst as part of its defense response. Antioxidants neutralize excess ROS produced during this response, preventing damage to the plant’s own cells [39,46].

Plants are exposed to a range of environmental conditions, and their ability to adapt is crucial for survival. Antioxidants play a role in helping plants adapt to changing environmental factors such as temperature fluctuations, drought, and nutrient imbalances. They mitigate the negative effects of these stressors on plant cells. Antioxidants protect the chloroplasts and other cellular structures involved in photosynthesis from oxidative damage [47]. Photosynthesis is a fundamental process for plant growth, and any disruption due to oxidative stress can have detrimental effects on overall plant health and productivity. Also, antioxidants contribute to delaying senescence, the aging process in plants. By scavenging free radicals and preventing cellular damage, antioxidants help extend the functional life of plant cells and tissues. This is particularly important for perennial plants that need to maintain vitality over multiple growing seasons [39,43,48].

**Figure 1:**
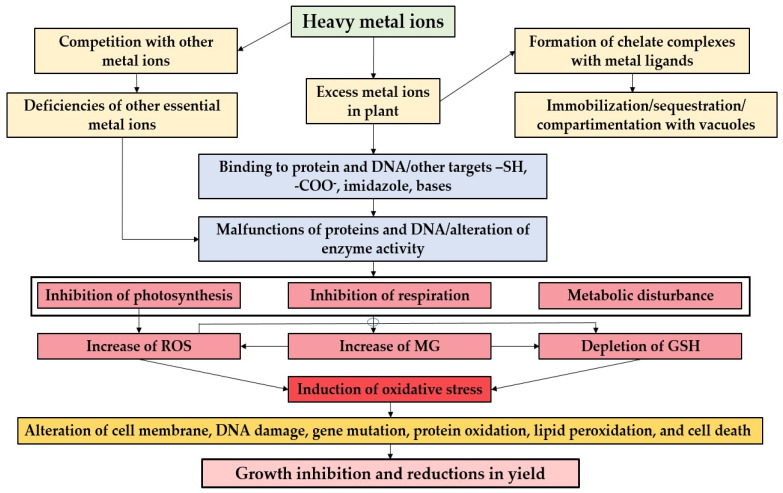
Possible biochemical and molecular mechanisms of heavy metal-mediated ROS induction and damage to the development of higher plants (reproduced upon Hossain et al. (2012), [50] under the Creative Commons Attribution License (CC BY), which permits unrestricted use, distribution, and reproduction in any medium, provided the original work is properly cited).

Antioxidants play a role in preserving the viability of seeds. They protect seeds from oxidative damage during storage and ensure that the seed’s genetic material remains intact. This is decisive for the successful germination and the growth of new plants. Antioxidants also participate in the regulation of cellular signaling pathways. They can influence gene expression and signaling cascades, helping plants respond to internal and external stimuli [46,49]. This regulatory role is essential for coordinating various physiological processes in the plant (Figure 1) [50].

### 2.3. Significance of Natural Antioxidant Activity in Maintaining Human Health

Antioxidant activity plays a paramount role in maintaining human health by counteracting the harmful effects of oxidative stress, a physiological imbalance between reactive oxygen species (ROS) and the body’s ability to neutralize them. Antioxidants contribute to the prevention and management of various diseases, including cardiovascular diseases, diabetes, and neurodegenerative disorders. They act as preventive agents and, in some cases, may complement medical treatments [6,39].

Oxidative stress is implicated in various health conditions and aging processes. The significance of antioxidant activity in human health encompasses several key aspects. Antioxidants protect cells from damage caused by free radicals, unstable molecules that can harm cellular structures, including DNA, proteins, and lipids. By neutralizing free radicals, antioxidants help maintain the integrity of cells, preventing mutations and reducing the risk of chronic diseases such as cancer. Antioxidants contribute to a robust immune system by safeguarding immune cells from oxidative damage. This ensures the proper functioning of immune responses, allowing the body to defend itself against infections, viruses, and other diseases more effectively [51].

Oxidative stress is a major contributor to cardiovascular diseases. Antioxidants help maintain cardiovascular health by protecting blood vessels from damage, reducing inflammation, and preventing the oxidation of low-density lipoprotein (LDL) cholesterol, a key factor in atherosclerosis. The brain is highly susceptible to oxidative stress, and its consequences are associated with neurodegenerative diseases such as Alzheimer’s and Parkinson’s. Antioxidants cross the blood-brain barrier, where they help protect neurons, modulate signaling pathways, and potentially mitigate cognitive decline. Chronic inflammation is linked to numerous health issues, including autoimmune diseases and cancer. Antioxidants exhibit anti-inflammatory properties by quenching free radicals and modulating inflammatory signaling pathways. This can help reduce the risk of chronic inflammatory conditions [43].

Antioxidants play also a decisive role in maintaining skin health and preventing premature aging. They counteract oxidative damage caused by UV radiation, pollution, and other environmental stressors, contributing to the preservation of skin elasticity, hydration, and overall youthful appearance. Antioxidants contribute to cancer prevention by neutralizing free radicals that can initiate DNA mutations and promote the development of cancer cells. While research on specific antioxidants and cancer risk is ongoing, a diet rich in antioxidants is generally associated with a lower risk of certain cancers [39,51,52,53].

### 2.4. Factors Affecting the Content of Antioxidants in Medicinal Plants

The medicinal properties of plants are often attributed to their rich content of bioactive compounds, particularly antioxidants. However, the content and activity of antioxidants in these plants can be influenced by various factors, both positively and negatively.

#### 2.4.1. Factors with Positive Influence on the Content of Antioxidants in Medicinal Plants

The environmental conditions such as geographical location, climate, and soil composition significantly impact the antioxidant content of medicinal plants. For instance, plants grown in regions with abundant sunlight and nutrient-rich soil tend to exhibit higher antioxidant levels [54,55]. Optimal environmental conditions contribute to the synthesis of secondary metabolites, including antioxidants. Proper agricultural practices, including organic farming and sustainable cultivation techniques, can enhance the antioxidant content in medicinal plants [56]. Adequate irrigation, balanced fertilization, and natural pest control methods contribute to healthier plants with increased antioxidant activity. The stage of plant maturity and the timing of harvesting play key roles in determining antioxidant levels.

In many cases, the concentration of antioxidants peaks during specific growth phases. Harvesting at the right time ensures maximal bioactive compound content, positively influencing the medicinal value of the plant [43]. The genetic diversity within plant species contributes to variations in antioxidant profiles. Selective breeding or genetic engineering approaches that emphasize antioxidant-rich varieties can be employed to enhance the overall therapeutic potential of medicinal plants.

#### 2.4.2. Factors with Negative Influence on the Content of Antioxidants in Medicinal Plants

Adverse environmental conditions, such as extreme temperatures, pollution, and exposure to harmful radiation, can negatively affect antioxidant content in medicinal plants [57]. These stressors may lead to an overproduction of reactive oxygen species (ROS), overwhelming the plant’s antioxidant defense mechanisms. Inadequate storage and processing methods can result in the degradation of antioxidants [35]. Exposure to light, air, and improper temperature during storage, as well as harsh processing techniques, may lead to the loss of bioactive compounds and a reduction in antioxidant activity. Continuous cultivation without proper soil management can deplete essential nutrients and minerals from the soil, impacting the synthesis of antioxidants in plants. Soil enrichment practices are essential in maintaining optimal nutrient levels for enhanced antioxidant production. Pest attacks and diseases can compromise the antioxidant content of medicinal plants. Plant responses to such stress often involve the redirection of resources away from antioxidant production, leading to a decline in overall bioactive compound levels [58].

Understanding and addressing these factors are essential for optimizing the medicinal potential of plants. By implementing proper cultivation, harvesting, and processing practices, as well as considering the environmental context, researchers and cultivators can enhance the antioxidant content and activity of medicinal plants, ultimately improving their therapeutic efficacy.

## 3. Impact of Environmental Pollution on Antioxidant Content and Activity in Medicinal Plants

Environmental pollution, resulting from various anthropogenic activities, poses a significant threat to the integrity of ecosystems and the health of living organisms. Pollutants such as heavy metals, pesticides, and industrial chemicals can accumulate in the soil and water where medicinal plants are grown. These contaminants can be absorbed by the plant and stored in its tissues, potentially altering its chemical composition and reducing its health benefits [57,59].

Medicinal plants, known for their rich array of bioactive compounds, including antioxidants, are particularly vulnerable to the adverse effects of environmental pollutants. In addition to affecting the chemical composition, pollution with pesticides and herbicides can contaminate the soil and water, and can also increase the levels of toxic compounds in the medicinal plants [5,54]. When these contaminated plants are consumed, they can cause a range of health problems, including cancer, neurological disorders, and reproductive issues [60,61].

Pollution can also have an impact on the availability and quality of medicinal plants. Many medicinal plants are grown in developing countries, where pollution is often more severe due to lax environmental regulations. This can result in a shortage of high-quality medicinal plants, making it more difficult for people to access these natural remedies [59]. This section explores how environmental pollution can influence the content and activity of antioxidants in medicinal plants.

### 3.1. Air Pollution

**Figure 2:**
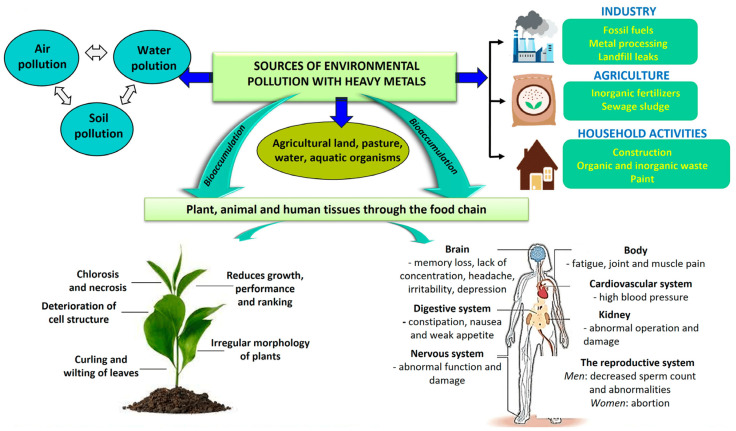
Sources and effects of heavy metals (Reproduced upon Vasilachi et al. (2023), [73] under the terms and conditions of the Creative Commons Attribution (CC BY) license (https://creativecommons.org/licenses/by/4.0/).

Air pollution refers to the presence of harmful substances in the air, which can be caused by a variety of sources. Some of the main sources of air pollution include industrial emissions, transportation, agriculture, and natural sources such as wildfires and dust storms (Figure 2). These pollutants can include particulate matter, ozone, nitrogen oxides, sulfur dioxide, and volatile organic compounds (VOCs) [62]. Airborne pollutants, such as particulate matter (PM) and heavy metals, can directly impact the antioxidant status of medicinal plants. The impact of air pollution on medicinal plants can be significant, as these plants can absorb pollutants from the air through their leaves and stems. Particles deposit on plant surfaces and enter through stomata, leading to increased oxidative stress. Heavy metals like lead, cadmium, and mercury disrupt enzymatic antioxidant defenses, thereby reducing the overall antioxidant activity in plants [63].

Tropospheric ozone and nitrogen dioxide, common air pollutants, can induce oxidative damage in plant tissues. This oxidative stress often leads to alterations in antioxidant metabolism, resulting in decreased levels of antioxidants. Ozone, in particular, interferes with photosynthesis, contributing to a decline in the synthesis of antioxidant compounds [64]. Exposure to nitrogen oxides can reduce the levels of flavonoids and phenolic acids in plants, which are important antioxidants and have been shown to have anti-inflammatory properties.

### 3.2. Water Pollution

Water pollution refers to the presence of harmful substances in water, which can be caused by a variety of sources. Some of the main sources of water pollution include industrial activities, agricultural practices, improper waste disposal, and natural sources such as erosion and runoff (Figure 2). Contamination of water bodies with industrial and agricultural runoff introduces pollutants such as pesticides, herbicides, and heavy metals.

The impact of water pollution on medicinal plants can be significant, as these plants can absorb pollutants from water through their roots [59,65]. Exposure to water pollutants can alter the chemical composition of medicinal plants, reducing their therapeutic properties. For example, exposure to heavy metals such as cadmium, mercury, and lead can reduce the levels of antioxidants and other medicinal compounds in plants. Pesticides, in particular, can disrupt the plant’s antioxidant defense system (Fan et al., 2023; Aye et al., 2019) [5,66]. Toxins present in polluted water, such as cyanotoxins and organic pollutants, can have detrimental effects on antioxidant metabolism in medicinal plants. The presence of these toxins can lead to cellular damage, prompting the plants to allocate resources away from antioxidant production in an attempt to counteract the immediate stress.

### 3.3. Soil Pollution

Soil pollution is a major concern around the world, as it can have a significant impact on human health and the environment. One area where soil pollution can have a particularly significant impact is on medicinal plants, which are valued for their therapeutic properties [54]. Soil contaminated with heavy metals, industrial chemicals, and pollutants from urban areas (Figure 2) negatively impacts the antioxidant content in medicinal plants [67]. The roots of these plants may absorb contaminants, leading to disruptions in antioxidant synthesis and an overall decrease in antioxidant activity. In addition to altering the chemical composition of medicinal plants, soil pollution can also reduce their growth and development. This can make it more difficult to cultivate these plants and can lead to a decrease in the availability of medicinal plants for use in traditional medicine and pharmaceuticals. The impact of soil pollution on medicinal plants can vary depending on the type of pollutant, the concentration of the pollutant, and the duration of exposure. For example, exposure to high levels of pesticides and herbicides can reduce the growth of medicinal plants and decrease the levels of important medicinal compounds such as alkaloids and terpenoids [2,48,68].

Pollutants can alter the composition and function of soil microbial communities, affecting nutrient cycling and availability. Changes in microbial activity can influence the symbiotic relationships between plants and soil microorganisms involved in antioxidant synthesis, thereby compromising the plants’ ability to produce antioxidants [69]. Prolonged exposure to environmental pollution can have cumulative effects on the antioxidant content of medicinal plants over time. As the levels of antioxidants decrease, the therapeutic efficacy of these plants may be compromised, potentially affecting the quality and effectiveness of herbal medicines derived from them.

Understanding the complex interplay between environmental pollution and antioxidant dynamics in medicinal plants is decisive for mitigating the impact on plant health and the potential therapeutic benefits derived from these plants. Strategies aimed at reducing pollution, implementing sustainable agricultural practices, and monitoring the quality of ecosystems are essential for preserving the medicinal properties of these valuable plant resources [70].

In addition to reducing the efficacy of medicinal plants, pollutants can also pose a health risk to people who consume them (Figure 2). Contaminated plants can contain high levels of toxic compounds, which can cause a range of health problems, including cancer, neurological disorders, and reproductive issues.

## 4. Heavy Metal Pollution of the Environment and Concerns for Medicinal Plants

### 4.1. Source of Environmental Pollution with Heavy Metals

Heavy metal pollution is a significant environmental concern, posing threats to ecosystems and human health. Several sources contribute to the release of heavy metals into the environment. Understanding these sources is vital for developing effective strategies to prevent and mitigate contamination. There are some key sources of heavy metal contamination (Figure 2).

Natural events, such as volcanic eruptions, can release heavy metals into the atmosphere. While volcanic activity is a natural source, it can contribute to localized contamination [47]. Natural weathering of rocks and minerals can release trace amounts of heavy metals into the soil and water. Certain geological formations may contain naturally elevated levels of metals.

Urban areas with high traffic volumes contribute to heavy metal contamination through the wear and tear of vehicle parts, road surfaces, and infrastructure. Stormwater runoff can carry these contaminants into rivers and streams [71]. Industrial activities such as mining and smelting operations are major contributors to heavy metal pollution. Activities such as extracting metals from ores and processing them release significant amounts of metals like lead, mercury, cadmium, and arsenic into the environment [60]. Various industries, including metallurgical, chemical, and electronic manufacturing, discharge heavy metals as byproducts. Effluents, emissions, and waste disposal from these processes contribute to environmental contamination. Accidental spills, leaks, or discharges from industrial facilities, such as chemical plants and mines, can result in sudden and significant releases of heavy metals into the environment [72]. Activities such as demolition and construction can disturb soil and release previously buried heavy metals. Construction materials, like paints and treated wood, may also contain heavy metal additives.

Agricultural practices are important sources of heavy metals in soil. The use of fertilizers and pesticides containing heavy metals, such as cadmium and mercury, can lead to soil contamination [5]. Agricultural runoff may carry these metals into water bodies, affecting both terrestrial and aquatic ecosystems. Livestock, when exposed to feed contaminated with heavy metals, can accumulate these metals in their tissues [74]. Manure from these animals, when used as fertilizer, can introduce heavy metals into soil and water.

Improper disposal of municipal and industrial waste, especially electronic waste (e-waste), can release heavy metals into landfills. Over time, these metals may leach into the surrounding soil and groundwater. Burning of waste materials, including medical waste and plastics, can also release heavy metals into the air and contribute to atmospheric deposition since they may then settle on land or water surfaces [75].

Atmospheric deposition can generate soil pollution. Industries and transportation activities release heavy metals into the atmosphere. Once airborne, these metals can travel over long distances before settling on land or water surfaces through rainfall or atmospheric deposition [76].

Addressing heavy metal contamination requires a multifaceted approach, including stringent regulations, responsible waste management practices, and the development of cleaner technologies in various industries. Public awareness and education are also essential to encourage sustainable practices and reduce the overall environmental impact of heavy metal sources.

### 4.2. Uptake and Accumulation of Heavy Metals in Medicinal Plants

#### 4.2.1. Exploring How Medicinal Plants Absorb and Accumulate Heavy Metals

Medicinal plants, well-regarded for their therapeutic properties, are not immune to the ubiquitous issue of heavy metal contamination in the environment. These plants, which often serve as vital components of traditional and modern medicine, can absorb and accumulate heavy metals through complex physiological processes. The exploration of how medicinal plants absorb and accumulate heavy metals is a topic of significant importance, particularly in the context of increasing environmental pollution. One of the key areas that require further exploration is the specific mechanisms through which medicinal plants absorb heavy metals. While it is acknowledged that these plants have the ability to absorb heavy metals from the environment, the exact processes involved in this absorption are not fully understood. Understanding how medicinal plants interact with and accumulate heavy metals is essential for assessing the potential risks associated with their use in herbal remedies.

Studies and research have identified some routes and mechanisms by which plants absorb and accumulate heavy metals [77,78,79,80]. The primary route for heavy metal entry into medicinal plants is through the roots. Plants absorb water and essential nutrients, but alongside these nutrients, they may inadvertently take up heavy metals present in the soil. The uptake of heavy metals by plant roots is influenced by factors such as soil pH, metal speciation, and the concentration of competing ions. Once absorbed by the roots, heavy metals can move within the plant through its vascular system. The xylem transports water and dissolved substances from the roots to the shoots, potentially carrying heavy metals along with essential nutrients. This upward translocation is influenced by the plant’s physiology and the mobility of specific metals [81,82].

Heavy metals may accumulate in various plant parts, with different plants exhibiting varying patterns of accumulation. Some medicinal plants tend to accumulate heavy metals in specific tissues or organs, such as leaves, stems, or roots. The accumulation pattern depends on the metal’s chemical properties and the plant’s physiology [66].

Another area that warrants further investigation is the defense systems that plants use to cope with the overproduction of reactive oxygen species (ROS) caused by heavy metals. While it is known that plants have enzymatic and non-enzymatic anti-oxidative defense systems, a more detailed exploration of these systems could provide valuable insights into how plants manage the stress caused by heavy metal exposure. Within plant cells, heavy metals may be bound to proteins, such as metallothioneins, or chelated by compounds like phytochelatins. These mechanisms serve as the plant’s defense strategy, sequestering and reducing the toxicity of heavy metals [83,84]. However, the effectiveness of these mechanisms varies among plant species. Exposure to heavy metals induces stress responses in plants, leading to alterations in gene expression and metabolic pathways [49]. These responses may include the synthesis of antioxidants and other compounds aimed at mitigating the toxic effects of heavy metals. The production of phytochelatins and glutathione are common responses to heavy metal stress [85,86].

Different medicinal plant species exhibit varying capacities for heavy metal uptake and accumulation. Some species may have a higher tolerance or resistance to certain metals, while others may be more prone to accumulation. This variability underscores the importance of considering plant species when assessing heavy metal contamination. The impact of heavy metal contamination on the biosynthesis of secondary metabolites in medicinal plants is another area that needs more research [3,87]. Changes in the quantity and quality of these compounds could potentially affect the medicinal properties of these plants. Therefore, understanding how heavy metal contamination influences the biosynthesis of secondary metabolites is essential [88,89].

The bioaccumulation of heavy metals in plants and the influence of environmental factors on this process is another area that requires further study. A comprehensive analysis of the interaction between plants and heavy metals could provide a better understanding of heavy metal tolerance in plants and could potentially lead to the development of more effective phytoremediation strategies [90,91]. The properties of the soil, including its texture, pH, and organic matter content, play a key role in the bioavailability and uptake of heavy metals by medicinal plants. Acidic soils, for instance, may enhance the solubility and uptake of certain metals. The bioavailability of heavy metals is influenced by their chemical forms in the soil. Some forms are more readily absorbed by plant roots than others. Soil conditions, such as redox potential, also affect the transformation of metals into more bioavailable forms [92].

Furthermore, there is a need for more research to establish scientific evidence criteria for herbal medicines. This is particularly important in the context of heavy metal contamination, as the safety and efficacy of these medicines could be compromised by the presence of heavy metals. Understanding the mechanisms by which medicinal plants absorb and accumulate heavy metals is vital for ensuring the safety and efficacy of herbal remedies [93]. Regular monitoring of heavy metal levels in both medicinal plants and the surrounding environment, coupled with sustainable cultivation practices, can help mitigate the risks associated with heavy metal contamination in medicinal plants [94]. This knowledge contributes to the development of guidelines and regulations to safeguard the quality and safety of herbal medicines.

#### 4.2.2. Factors Influencing the Heavy Metals Uptake Process by Herbs

The uptake of heavy metals by plants is a complex and dynamic process influenced by various factors that span both the environmental and plant physiological ranges. Understanding these factors is essential for comprehending the complexities of metal uptake and for developing strategies to manage and mitigate potential contamination. Some key factors influencing the uptake process are discussed in Table 1.

Considering these factors in the context of metal uptake by plants is essential for managing and remediating contaminated environments. Research on the interactions between these factors continues to contribute to the development of sustainable practices for reducing the risks associated with heavy metal exposure in both agricultural and natural ecosystems, in particular exploitation of medicinal plants.

### 4.3. Impact of Heavy Metals on Medicinal Plant Compounds

#### 4.3.1. Heavy Metals Impact on the Synthesis and Concentration of Medicinal Plant Compounds

**Figure 3:**
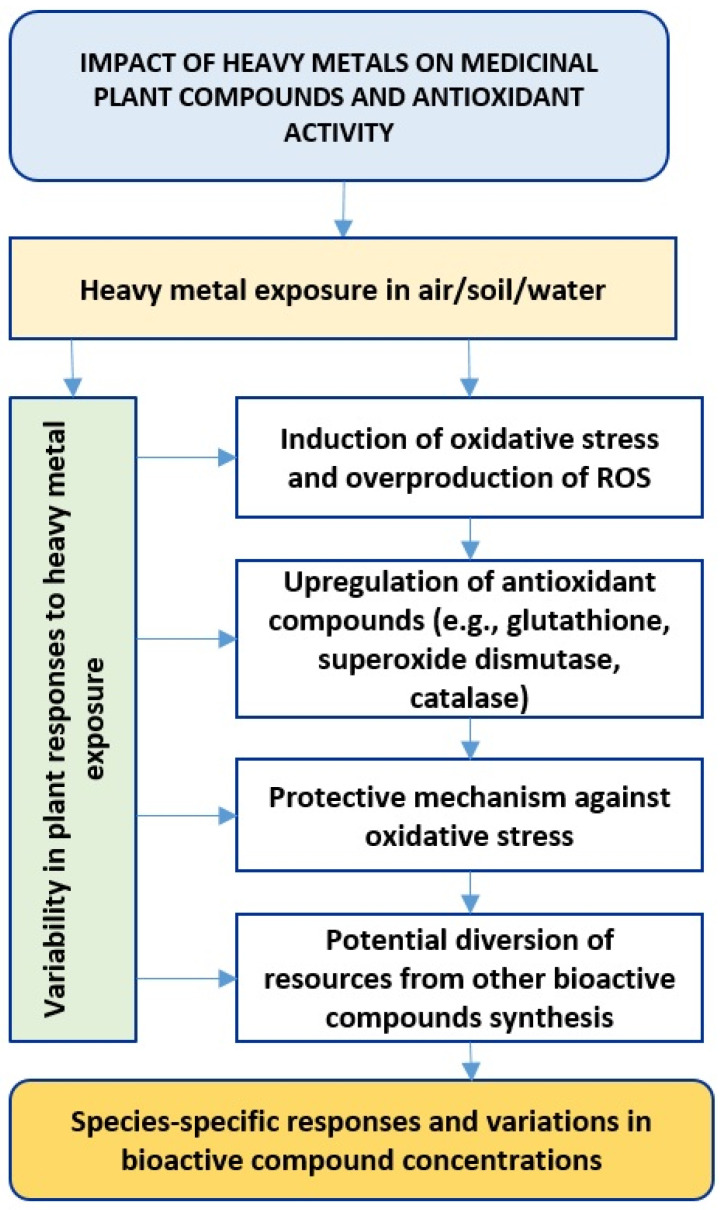
Impact of heavy metals stress on medicinal plant compounds and antioxidant activity.

Exposure to elevated levels of heavy metals, such as cadmium, lead, and mercury, has been found to have adverse effects on medicinal plants. Starting from heavy metal exposure in soil or water, this leads to variability in plant responses, during induction of oxidative stress, upregulation of antioxidant compounds, and potential diversion of resources [113,114,115]. The ultimate result is species-specific responses and variations in the concentrations of bioactive compounds in medicinal plants (Figure 3).

Various studies suggest that the synthesis of secondary metabolites, essential for the therapeutic properties of these plants, is negatively impacted under such conditions (Figure 3). Alkaloids, flavonoids, and phenolics, which fall under the category of secondary metabolites, may experience reduced synthesis [4,67,116]. Furthermore, heavy metal stress is linked to decreased concentrations of specific bioactive compounds. For instance, plants exposed to heavy metal contamination exhibit lower concentrations of flavonoids and polyphenols [117,118].

The presence of heavy metals in medicinal plants can alter metabolic pathways in two key ways [10,119]. Firstly, heavy metals can disrupt various biosynthetic pathways within plants, leading to changes in the production of secondary metabolites. This disruption may particularly affect the synthesis of compounds like antioxidants and antimicrobial agents. Secondly, heavy metal stress can impact the activity of enzymes involved in the biosynthesis of bioactive compounds, contributing to observed alterations in the concentrations of secondary metabolites [3,13,120].

Exposure to heavy metals in medicinal plants has significant implications, notably in inducing oxidative stress and triggering antioxidant responses. This occurs through two key mechanisms. Firstly, heavy metal exposure often leads to the induction of oxidative stress within plants. This stress results in the overproduction of reactive oxygen species (ROS), which, in turn, negatively impacts cellular structures and metabolic processes. Secondly, in response to oxidative stress, plants may employ a protective mechanism by upregulating the synthesis of antioxidant compounds. These compounds include glutathione, superoxide dismutase, and catalase. While serving as a defense against oxidative stress, this process may divert resources away from the synthesis of other bioactive compounds [6,121].

The effects of heavy metal presence in medicinal plants are not uniform and depend on species-specific responses. Variability among different plant species is observed, with some demonstrating a higher tolerance to heavy metals, allowing them to maintain the production of certain secondary metabolites [67,87]. In contrast, others may experience more pronounced changes.

Another nuanced aspect of heavy metal impact on herb potency involves medicinal plant adaptations. Some plants adopt a defense strategy by accumulating heavy metals. However, this accumulation may result in alterations in the concentrations of bioactive compounds. Additionally, variations in the accumulation of heavy metals in different plant tissues, such as roots accumulating higher concentrations compared to above-ground parts, can influence the distribution of bioactive compounds within the plant.

Moreover, the environmental conditions, particularly soil properties, influence the synthesis and concentration of medicinal plant compounds in a complex way. Factors such as soil pH, organic matter content, and others (Table 1) play a role in determining metal bioavailability and subsequent effects on plant metabolism [23,122].

While these findings offer valuable insights, it’s essential to acknowledge the complexity of interactions between heavy metals and medicinal plants. Ongoing research should be dedicated to deepening the understanding of these dynamics and exploring potential strategies to mitigate the adverse effects of heavy metal contamination on the quality and efficacy of medicinal plants.

#### 4.3.2. Variations in Responses among Different Medicinal Plant Species versus Heavy Metal Action

The responses of different plant species to heavy metal exposure exhibit considerable variations, reflecting the diverse strategies employed by plants to cope with metal stress. These variations are influenced by species-specific physiological, biochemical, and genetic factors [50,60,123]. The observed diversity in these responses underscores the dynamic and adaptive strategies that plants employ to contend with the challenges posed by metal stress. This variability is shaped by a combination of species-specific physiological, biochemical, and genetic factors, resulting in a nuanced complex of reactions across the plant kingdom. As the complex web of plant responses to heavy metal action is continuously explored, it becomes evident that no two species react in precisely the same way. Instead, a spectrum of adaptations and coping mechanisms unfolds, providing a remarkable insight into the resilience and versatility of plant life in the face of environmental stressors [59,121].

In an effort to shed light on the multifaceted nature of these responses, key aspects showcasing the diversity among different plant species in their reactions to heavy metal exposure have been documented in literature, as shown in Table 2, to help understanding the complex interplay between plant physiology, biochemistry, and genetics in the context of metal stress. Through a closer examination of these variations, it is possible to gain a deeper appreciation for the complex and species-specific dynamics that dictate how plants navigate the challenges posed by heavy metal presence in their environments [121].

Some of the interactions presented in Table 2 are described below, since they exert a decisive role in heavy metals impact on medicinal plants effectiveness. The unique attributes of each plant species contribute to the overall resilience and diversity observed in metal-contaminated environments. By unraveling these intricate interactions, we can better leverage the natural capabilities of plants to mitigate the effects of heavy metal pollution and foster environmental sustainability. Understanding these variations in responses among different plant species is essential for predicting the ecological consequences of heavy metal contamination and for developing strategies for phytoremediation, ecological restoration, and sustainable land management.

#### 4.3.3. Soil Microbial Interactions and Interactive Soil-Metal-Plant Relationships

In the soil environment, microbial communities are essential actors in moderating interactions between heavy metals and medicinal plants. Specifically, the rhizosphere, the region surrounding plant roots, serves as a vital interface for these interactions. Within this domain, soil microbes engage in processes that influence the mobility and availability of heavy metals, as well as the responses of medicinal plants to environmental stresses. Through symbiotic relationships and metabolic pathways, soil microbes contribute significantly to the dynamic relationships between soil, metals, and plants. Understanding these microbial interactions provides valuable insights into the relationships between soil, heavy metals, and medicinal plants, offering strategies for sustainable cultivation and environmental management:

*(i) Rhizosphere microbiome:* Rhizosphere interactions are revealed as critical facets of the dynamic interaction between plant roots and soil microorganisms. This zone, where complex connections between plants and their microbial companions occur, assumes a central role in influencing various aspects of plant well-being. Notably, beneficial microbes within the rhizosphere contribute significantly by enhancing nutrient availability, participating in the immobilization of heavy metals, and influencing the activation of plant defense mechanisms [135]. Within the rhizosphere, the soil microbiome assumes a critical role in orchestrating interactions between medicinal plants and heavy metals. This microbial community, complexly associated with the plant’s root system, becomes a key mediator in influencing the plant’s response to heavy metal stress. Beneficial microbes dwelling in the rhizosphere contribute to the plant’s resilience by facilitating nutrient uptake, modulating stress responses, and participating in the breakdown of metal complexes in the soil [69,134]. The dynamic interaction between the rhizosphere microbiome and medicinal plants helps us understand the complex mechanisms by which microbial interactions influence the plants ability to thrive in metal-contaminated environments [133].

*(ii) Mycorrhizal associations:* Mycorrhizal symbioses form another critical dimension of soil microbial interactions that impact heavy metal-plant dynamics. Mycorrhizal associations, a noteworthy component of these interactions, exhibit the capacity to amplify the plant’s tolerance to heavy metals and modulate its antioxidant responses [133]. These associations exert influence over the uptake of heavy metals, playing a role in modulating the antioxidant responses of medicinal plants. Mycorrhizal associations contribute to metal tolerance by enhancing the plant’s ability to cope with metal stress. Additionally, they may alleviate oxidative stress within the plant, ultimately contributing to its overall fitness in challenging environmental conditions [136,137].

Communication between plants and rhizospheric microorganisms involves signaling molecules. Plant-microbe interactions influence the expression of genes related to antioxidant defense, metal uptake, and stress response pathways. Within this belowground zone, communication between plants and rhizospheric microorganisms becomes an interesting process mediated by signaling molecules. This communication, often refined but impactful, encompasses plant-microbe interactions that extend beyond the visible spectrum [134,138]. The consequences of these interactions resonate in the expression of genes related to antioxidant defense mechanisms, metal uptake processes, and the activation of stress response pathways within plants. In essence, the rhizosphere serves as a dynamic platform where the language of molecular signaling governs the cooperative interactions between plants and their underground microbial counterparts, ultimately influencing the plant’s ability to navigate challenges presented by metal stress [133].

Understanding the details of these interactions provides valuable insights into the collaborative strategies employed by soil microbes and medicinal plants in crossing the complexities of heavy metal-containated soil.

#### 4.3.4. Hormonal Regulation

In plant stress responses, hormones play a decisive role. When plants encounter heavy metals, their hormone levels change, affecting how they produce antioxidants and other helpful substances. This change adds complexity to how plants deal with metal stress. Also, these hormones influence which genes are turned on or off, helping plants adapt to metal stress by adjusting how they respond to harmful substances.

Understanding how hormones and genes work together is important to be studied further so as to cover all features, since it can give important evidences about how plants cope with metal stress:

*(a) Phytohormone signaling:* The regulatory framework of plant stress responses is complexly interconnected with phytohormones, including abscisic acid (ABA) and jasmonic acid (JA). Under the influence of heavy metals, these hormonal signaling pathways become modulated, impacting the synthesis of antioxidant enzymes and secondary metabolites within medicinal plants [120,139]. The complex exchange between phytohormones adds a layer of complexity to the plant’s adaptive mechanisms, as heavy metal-induced alterations in hormonal networks influence the overall biochemical and physiological responses of the plant to metal stress [140,141].

*(b) Stress-responsive genes:* Hormonal regulation extends its influence to the expression of stress-responsive genes, constituting a tightly orchestrated network of molecular responses within medicinal plants. Heavy metals induce changes in gene expression patterns, activating or suppressing specific genes involved in antioxidant defense [142,143]. This dynamic modulation of stress-responsive genes shapes the adaptive responses of the plant to metal stress, fine-tuning its capacity to cope with oxidative challenges. Understanding the complex relationship between hormonal signaling and gene expression provides critical insights into the regulatory mechanisms governing the adaptive responses of medicinal plants to heavy metal-induced stress.

#### 4.3.5. Epigenetic Modifications and Regulation

The exposure of medicinal plants to heavy metals can cause important changes in how their genes work, specifically through processes involving DNA methylation and histone modifications. These changes affect the plants ability to deal with stress and can have long-lasting effects. Heavy metals, by altering the plant genetic structure, influence its response to challenges like oxidative stress. In the context of epigenetic regulation, heavy metal-induced stress initiates a cascade of molecular events resulting in modifications that transcend the genetic code. What’s more, these effects go beyond the immediate generation, impacting future plant populations in metal-contaminated areas. Understanding these genetic changes provides essential insights into how heavy metals affect medicinal plants over time, influencing their ability to adapt and thrive.

*(a) DNA methylation and histone modifications:* Advancements in research indicate that heavy metal exposure can instigate epigenetic alterations in medicinal plants. This phenomenon, manifested through DNA methylation and histone modifications, becomes a pivotal mechanism altering the expression patterns of genes associated with antioxidant systems within medicinal plants [144,145]. The regulatory influence of these epigenetic modifications extends beyond immediate responses, shaping the long-term adaptive trajectories of plants in the face of heavy metal stress [49,61]. Specifically, DNA methylation and histone modifications may undergo changes, influencing the expression of genes associated with antioxidant pathways [61]. This molecular restructuring provides a nuanced understanding of the long-term impact of heavy metals on the responses of medicinal plants. By modulating the epigenetic landscape, heavy metals can exert regulatory control over gene expression, shaping the plant’s ability to navigate and respond to oxidative stress induced by metal exposure [49].

*(b) Transgenerational effects:* The implications of epigenetic modifications extend beyond immediate responses, with transgenerational effects becoming a noteworthy facet of heavy metal-plant interactions [146,147]. The heritability of epigenetic changes contributes to transgenerational responses to heavy metals, influencing the adaptability of plant populations over successive generations in metal-contaminated environments [142,143]. The induced changes in DNA methylation and histone modifications can be passed down from one generation to the next, imprinting a molecular memory within the plants genetic heritage. This transmission of epigenetic information holds the potential to perpetuate alterations in the antioxidant capacity of successive plant generations, particularly in environments fraught with metal contamination [148]. The enduring influence of epigenetic regulation on the adaptive responses of medicinal plants underscores the significance of considering not only immediate, but also transgenerational effects when interpreting the impacts of heavy metals on plant physiology.

Unraveling these transgenerational effects at the epigenetic level is essential for a comprehensive understanding of the enduring impact of heavy metals on medicinal plants and the potential implications for the overall resilience and adaptive capacity of plant populations.

#### 4.3.6. Interactive Effects with Environmental Factors

Medicinal plants face challenges from both heavy metals and air pollutants in their environment. Climate variations, like changes in temperature and rainfall, can affect how plants absorb and respond to metals. Similarly, air pollutants can interact with heavy metals, making the plant’s response even more complicated. These interactions influence how plants produce antioxidants and other helpful substances. Understanding how these environmental factors work together is important for thinking out how medicinal plants respond to stress and how they can be helped to stay healthy.

*(i) Climate and seasonal variations:* The influence of heavy metals on medicinal plants is subject to variations associated with climate and seasonal changes. Factors such as temperature, precipitation, and light intensity play critical roles in shaping the dynamics of metal uptake, translocation, and the subsequent antioxidant responses within the plant, as mentioned above, too [55,81].

Notably, seasonal variations emerge as influential factors, potentially impacting the bioavailability of metals and thereby influencing the dynamics of oxidative stress and the activation of antioxidant defense mechanisms [23,149].

The complex interaction between heavy metals and environmental factors underscores the need for a comprehensive understanding of how climatic and seasonal variations modulate the impact of metal exposure on medicinal plant physiology [76,149].

*(ii) Interactive effects with air pollutants:* As revealed above, medicinal plants often confront concurrent exposure to heavy metals and air pollutants, initiating a complex interplay between these environmental stressors. The combined effects of heavy metals and air pollutants may result in interactive responses within the plant, influencing both antioxidant systems and the synthesis of bioactive compounds [76]. This interactive dynamic adds a layer of complexity to the plants adaptive strategies, as it navigates the multifaceted challenges posed by the simultaneous presence of heavy metals and air pollutants in the environment [150]. Understanding these interactive effects is critical for unraveling the holistic responses of medicinal plants to environmental stressors and for devising strategies to mitigate the impact on their therapeutic potential.

## 5. Mechanisms of Heavy Metal-Induced Changes in Antioxidant Activity

The molecular and biochemical mechanisms underlying the impact of heavy metals on antioxidant activity in medicinal plants represent dynamic and complex of interactions. Straightening out these mechanisms can elucidate the adaptive strategies employed by medicinal plants, to face the toxic effects of heavy metals as pollutants. As research continues to advance, a deeper understanding of these mechanisms will contribute to the development of strategies to mitigate the adverse effects of heavy metal contamination on medicinal plants and ensure their continued role in healthcare and traditional medicine.

### 5.1. Molecular and Biochemical Insights into the Impact of Heavy Metals on Antioxidant Activity in Medicinal Plants

**Figure 4:**
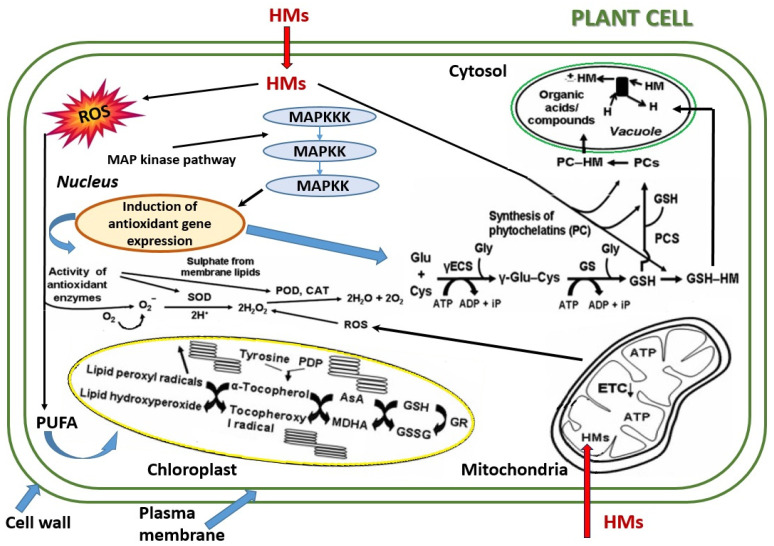
Development of oxidative stress in plant cells due to the presence of heavy metals, triggering various mechanisms of tolerance and detoxification (these processes involve the use of important molecules and enzymes such as ADP-adenosine dinucleotide phosphate, As-Aascorbic acid, ATP-adenosine triphosphate phosphate, CAT-catalase, Cys-cysteine, c-ECS-c-glutamylcysteine synthetase, ETC- electron transport chain damage, Glu-glutamine, Gly-glycine, GR-glutathione reductase, GS-glutathione synthetase, GSH-glutathione (reduced), GSSG-glutathione (oxidized), H-hydrogen molecule, HMs-heavy metals, H_2_O_2_-hydrogen peroxide, MAPK-mitogen-activated protein kinase, MAPKK-MAPK kinase, MAPKKK-MAPK kinase kinase, MDHA-monodehydroascorbate, iP-inorganic phosphate, O_2_-oxygen molecule, O_2_^•−^ superoxide radicals, PCs-phytochelatins, PC-phytochelatins, PDP-phytol diphosphate, POD- peroxidases, PUFA-polyunsaturated fatty acids, ROS-reactive oxygen species, SOD-superoxide dismutase, SQDG-sulfoquinovosyldiacyglycerol) (adapted upon Sytar et al., 2011 [153]. Reused with permission from the Publisher, License number 5751980234761, from 18 March 2024).

Medicinal plants, valued for their therapeutic properties, are not immune to the persistent influence of heavy metal contamination in the environment. The complex interaction between heavy metals and the antioxidant activity of medicinal plant compounds unfolds at the molecular and biochemical levels, revealing a complex network of interactions that shape the plants ability to produce vital bioactive molecules [122]. Metal exposure can lead to noticeable signs of phytotoxicity in sensitive medicinal plants. Common manifestations of heavy metal toxicity include the production of reactive oxygen species (ROS) and reactive nitrogen species (NO), as well as the disruption of enzyme cofactors and transcription factors [120]. This can also result in the inhibition of antioxidative enzymes, imbalances in cellular redox levels, disruptions in ionic transport, DNA damage, and protein oxidation [86,116]. The stress from heavy metals causes a decrease in molecular oxygen and the production of harmful intermediate products such as superoxide radicals (O_2_^•−^), hydroxyl radicals (OH^•−^), and hydrogen peroxide (H_2_O_2_). These active molecules can have a more toxic and reactive effect than oxygen, leading to chain reactions in the membrane lipids and proteins, ultimately causing oxidative damage (Figure 4) [151,152]. It is essential to explore the mechanisms underlying this impact, providing a nuanced perspective on the molecular responses of medicinal plants to heavy metal stress.

#### 5.1.1. Heavy Metal Uptake and Translocation

In the complex dynamics of medicinal plant physiology, a fundamental part reveals as resilient herbs engage in a dynamic interaction with their surroundings. As mentioned above, at the core of this process lies the root system, serving as the initial gateway for heavy metals impact into the plants physiology. During this important step of metal absorption, a complex system of transporters and channels guides the process, acting like gatekeepers to help metal ions enter the plant’s vascular system. In this arrangement, there are identified two mechanisms [66,107,108,126]:

*(1) Root absorption:* At the basis of medicinal plant physiology, the complex processes happens in the root system. Here, these resilient herbs engage in a dynamic interaction with their environment, absorbing heavy metals from the soil. This initial phase of metal uptake is coordinated and facilitated by a complex network of specialized transporters and channels, acting as gatekeepers that guide metal ions into the plants vascular system. The roots, acting as both keepers and channels, play a key role in shaping the fate of heavy metals within the medicinal plant. The transporters, which govern the influx of heavy metals into the plant, undergo particular regulation and expression, primarily mediated by sophisticated signaling pathways.

Among these pathways are those directed by mitogen-activated protein kinases (MAPKs) and calcium-dependent protein kinases (CDPKs), both instrumental in shaping the dynamics of heavy metal transport across the plant’s vascular system [154,155]. Concurrently, heavy metals serve as inducers for the generation of reactive oxygen species (ROS) within plant cells, setting off a cascade of signaling events. ROS, acting as secondary messengers, initiate intricate signaling cascades that extend beyond the limits of cellular boundaries [86]. This cascade includes the activation of various plant hormones, such as salicylic acid (SA), jasmonic acid (JA), and ethylene (ET). This interplay between heavy metals and ROS signaling pathways acts as a critical regulatory mechanism, guiding the plant’s responses to the challenges posed by metal exposure [139,156]. It also significantly influences the activation of hormone-mediated signaling cascades, thus contributing to the overall adaptive responses of the plant to heavy metal stress.

*(2) Translocation to shoots:* As the absorbed heavy metals get on on their botanical passage, they traverse the complicated network of vascular tissues within the plant. This passage culminates in the raise of these metal ions, reaching the aerial region of leaves and stems. The translocation to shoots is a critical stage, holding the key to the subsequent impact on the plants biochemical pathways. The selective distribution of heavy metals within these aerial structures becomes a strategic determinant, influencing the overall health and therapeutic potential of the medicinal herb.

In order to assess the capacity of plants in accumulating metal, calculations were performed for the bioaccumulation coefficient factor (BCF) and translocation factor (TF). BCF is determined by dividing the metal concentration in the root by the concentration in the soil, while TF is determined by dividing the metal concentration in the shoot by the concentration in the root [157,158]. These calculations, previously reported by Yoon et al. (2006) [159], were carried out using Equations (1) and (2):

BCF = C_root_/C_soil_ (1)

TF = C_shoot_/C_root_ (2)

where: C_root_ represents the metal concentration in the plant’s root (mg kg^−1^), C_soil_ represents the metal concentration in the distillery sludge (mg kg^−1^), and C_shoot_ is the metal concentration in the plant’s shoot per dry weight (mg kg^−1^). It is important to consider both BCF and TF when determining if a plant is capable of hyperaccumulating metal.

In the process of absorbing and moving nutrients, medicinal plants show how they can adapt to tough environments. They carefully manage getting important nutrients while dealing with the possible harm from heavy metals. Thinking on these complicated processes is crucial for understanding how medicinal plants interact with their environment. It helps us find better ways to grow herbs sustainably and make sure they keep working effectively as natural remedies.

#### 5.1.2. Oxidative Stress Initiation

Examining how heavy metals initiate oxidative stress in medicinal plants is crucial. It sets the groundwork for understanding how these plants handle metal challenges and why reactive oxygen species (ROS) become active. It also sheds light on why parts of plant cells, such as mitochondria, are sensitive to heavy metals. Exploring the fundamentals of how heavy metals cause stress in herbs opens avenues for learning more about plant adaptation. Two mechanisms merit discussion in this context (Figure 4) [86,153,160]:

*(a) Reactive Oxygen Species (ROS) generation:* As heavy metals infiltrate cellular compartments, a cascade of events is set in motion within the complex equipment of plant cells. At the heart of this process is the initiation of reactive oxygen species (ROS) generation. Heavy metals, acting as catalysts, induce the formation of ROS, including superoxide radicals and hydrogen peroxide. These molecular entities play dual roles as both signaling molecules and instigators of cellular damage, setting the stage for oxidative stress within the plant [44,45,83,84].

*(b) Mitochondrial dysfunction:* Among the cellular organelles susceptible to the disruptive influence of heavy metals, the mitochondria stand as key players in the process of oxidative stress. These essential energy-producing structures become particularly vulnerable to the stress induced by heavy metals. Dysfunction in the electron transport chain, a critical process within the mitochondria, can lead to the leakage of electrons. This leakage contributes to the heightened production of ROS, thereby intensifying the oxidative stress experienced by the plant. The delicate balance within the mitochondria, essential for cellular energy production, is perturbed, reverberating the broader impact of heavy metal exposure on the plant’s overall well-being [154,160,161].

In this perspective of oxidative stress initiation, the interaction between heavy metals and the plants cellular components reveals a relation of molecular complexities and biological responses. Straightening out these processes not only deepens the understanding of the physiological consequences of heavy metal exposure but also can reveal potential strategies for mitigating oxidative stress in medicinal plants, safeguarding their therapeutic properties and ecological resilience.

#### 5.1.3. Antioxidant Defense Mechanisms

The medicinal plants have the ability to protect themselves when facing heavy metal contamination, by a defense mechanisms these plants use to counteract the stress caused by metals. Enzymatic antioxidants like superoxide dismutase and non-enzymatic antioxidants like ascorbic acid play key roles. By understanding these defense strategies, it is possible to understand the mechanism herbs maintain their balance and resilience in the face of heavy metal challenges) [6]. These strategies include:

*(i) Enzymatic antioxidants:* In response to the imminent threat of heavy metal-induced oxidative stress, medicinal plants employ a sophisticated arsenal of enzymatic antioxidants [81,162]. At the forefront of this defense are enzymes such as superoxide dismutase (SOD), catalase (CAT), and peroxidases, each playing a critical role in neutralizing reactive oxygen species (ROS). SOD facilitates the dismutation of superoxide radicals, while CAT and peroxidases work in tandem to decompose hydrogen peroxide and other peroxides. Through these enzymatic activities, medicinal plants carefully manage their functions, capturing ROS and keeping the balance right within their cells [97,120,163,164].

*(ii) Non-enzymatic antioxidants:* Complementing the enzymatic defense mechanisms, medicinal plants strategically deploy non-enzymatic antioxidants as an essential line of defense against heavy metal-induced oxidative stress. Molecules like ascorbic acid, glutathione, and tocopherols play vital roles as they donate electrons, helping protect cells in a complex process [154]. For example, glutathione, as a key non-enzymatic antioxidant, key non-enzymatic antioxidant in the metal detoxification process, forms complexes with heavy metals, facilitating their detoxification within the cellular environment. This complex molecular interaction prevents heavy metal-induced damage to cellular components and actively contributes to maintaining a delicate balance in cellular redox equilibrium [86,165]. These non-enzymatic antioxidants play a key role in neutralizing ROS, intercepting their damaging potential, and preventing oxidative harm to vital cellular components. The harmonious interaction between enzymatic and non-enzymatic antioxidants showcases the resilience of medicinal plants in crossing the oxidative challenges imposed by heavy metal exposure [81,166].

In the antioxidant defense mechanisms, medicinal plants display proficient self-preservation, using various methods to fight against the harmful effects of heavy metals. Understanding these complex defense strategies not only reveals how resilient these plants are but also suggests new ways to boost their antioxidant abilities when facing environmental challenges.

#### 5.1.4. Metal-Chelating Compounds and Detoxification

Medicinal plants can respond to heavy metal stress by producing metal-chelating compounds. These compounds, like phytochelatins and metallothioneins, act as defenders, binding to heavy metals and preventing them from causing harm within the plant [83,84,125]. Understanding the role of these chelating compounds sheds light on the ingenious ways herbs shield themselves from the adverse effects of metal exposure.

Confronted with the imminent threat of heavy metal stress, medicinal plants disclose a sophisticated defense strategy through the synthesis of metal-chelating compounds, with *phytochelatins and metallothioneins* taking center stage. These remarkable peptides serve as guardians, coordinating a molecular movement to counteract the potential harm produced by heavy metals. Phytochelatins and metallothioneins, formed inside the cells of medicinal plants, possess a unique ability to bind tightly with heavy metals, forming stable complexes. This binding not only sequesters the metals but also shields the plants vital cellular biomolecules from the detrimental interactions that these metals may induce [83,111].

As natural chelators, phytochelatins and metallothioneins play important roles in the intricate relationship between medicinal plants and heavy metal stress. The synthesis of these metal-chelating compounds reflects the adaptive genius of these botanical healers, offering a biochemical shield against the potential toxicity of heavy metals [111].

Understanding the production of phytochelatins and metallothioneins within medicinal plants not only unravels the secrets of their resilience but also can encourages research for harnessing these natural defenders in the detection of sustainable strategies for mitigating the impact of heavy metal stress on plant health.

#### 5.1.5. Bioactive Compound Synthesis

Heavy metal stress can induce changes in the synthesis of bioactive compounds within medicinal plants. When faced with metal challenges, herbs may redirect their resources, affecting the production of compounds like polyphenols, alkaloids, and flavonoids [2]. These changes affect the quality and healing properties of herbs, helping in understanding how heavy metal stress influences the production of important bioactive compounds:

*(a) Diversion of resources:* In the face of heavy metal stress, medicinal plants undergo a profound reprogramming of their metabolic pathways, initiating a strategic response to cope with the impending threat. In this complicated survival process, plants often adjust where they allocate resources, focusing more on fighting oxidative stress [121]. Unfortunately, this reallocation may come at a cost, leading to the diversion of precursor molecules and energy away from the synthesis of bioactive compounds. Polyphenols, alkaloids, and flavonoids—as denoted above as key players in the therapeutic role of medicinal plants—may experience a decline in production as the plant prioritizes its resources to face the challenges posed by heavy metal exposure [114,117].

*(b) Gene expression alterations:* At the molecular level, heavy metal stress significantly affects the genetic processes responsible for producing bioactive compounds in medicinal plants. Genes involved in these pathways may change their activity in response to exposure to heavy metals in the environment [125,147]. These changes in gene expression, driven by transcriptional regulation, have a chain reaction on the levels of specific bioactive compounds. The dynamic interplay between the plants genetic structure and its response to heavy metal stress thus becomes a critical determinant, shaping the overall therapeutic potential of medicinal plants in the midst of environmental challenges [127,143].

Hence, when heavy metals affect how plants make bioactive compounds, medicinal plants adapt to the stress in a careful balance. By studying how resources are used and genes are activated, it is possible to discern the complex processes shaping the plants chemical behaviour under stress. This understanding can help in maintaining the plants’ medicinal properties and find sustainable ways to grow them despite changing environments.

#### 5.1.6. Species-Specific Responses

In the world of medicinal plants facing heavy metal exposure, each species reveals its unique response to the challenge. The genetic diversity among these medicinal plants shapes their ability to combat oxidative stress caused by heavy metals [60,125]. Differences in gene expression, genetic characteristics, and specific alleles lead to diverse reactions in various herb species. Studying these unique responses helps us understand how their genetic makeup affects their ability to survive in metal-contaminated environments [39]:

*(i) Genetic diversity:* The complex interaction between heavy metals and antioxidant activity in medicinal plants unfolds against the backdrop of genetic diversity inherent to each species. The impact of heavy metals is not a uniform experience, as variations in gene expression, the presence of specific alleles, and genetic traits related to metal tolerance contribute to the diverse responses observed among different plant species [97,121]. The detailed genetic structure of each medicinal plant species plays a critical role in shaping its capacity to endure and cope with the challenges of exposure to heavy metals. This, in turn, affects the plants antioxidant responses and overall outcomes [2,121].

*(ii) Phenotypic plasticity*: Within the range of heavy metal stress, some medicinal plant species showcase a remarkable ability for phenotypic plasticity—an adaptive trait that allows them to dynamically respond to changing environmental conditions. This plasticity is manifested in various ways, including alterations in leaf morphology, adjustments in root architecture, and shifts in antioxidant enzyme activities [167]. These phenotypic variations serve as tangible expressions of the plants resilience, influencing its overall capacity to thrive in metal-contaminated environments. As each species unfolds its unique set of adaptive responses, the complex interactions between genetic diversity and phenotypic plasticity becomes a defining feature in decoding the diverse strategies employed by medicinal plants in the face of heavy metal challenges [168].

In studying how different species respond uniquely, it is possible to discover the genetic specifics and physical changes of medicinal plants. This understanding not only enhances the knowledge of plant ecology, but also guides customized plans for preserving and growing these healing plants sustainably amidst environmental changes.

#### 5.1.7. Interactive Effects of Co-Contaminants

In the dynamic reality of herb ecosystems, contamination rarely comes in isolation. The interactive interaction of heavy metals and co-contaminants creates a complex scenario for these resilient plants. Simultaneous exposure to multiple pollutants shapes the fate of medicinal herbs. Whether co-contaminants exacerbate or mitigate the impact on antioxidant activity, the complex relationship of various stressors and their combined effects on herb responses cumulates [113,169,170]. Understanding the complexity of these effects involves the exploration of the interactive world of heavy metals and contaminants in herbs, such as synergistic or antagonistic interactions. The passage of medicinal plants through environmental landscapes often reveals the presence of multiple stressors, where co-contamination with various heavy metals or other pollutants becomes a reality. In this complex relation between plant and environment, the interactive effects of co-contaminants emerge as critical determinants in shaping the overall impact on antioxidant activity [171]. The dynamic interchange between these pollutants can lead to either synergistic collaborations, intensifying the stress on medicinal plants, or antagonistic interactions, offering a form of lessening by mitigating the detrimental effects [170]. This nuanced relationship between co-contaminants adds another layer of complexity to the plants responses, highlighting the need to explore not only individual stressors, but also their combined influence in real-world scenarios.

As medicinal plants deal with environmental challenges, it’s important to study how different contaminants interact. Understanding these interactions helps predict and manage how plants respond to pollution. This knowledge guides sustainable cultivation and conservation, preserving the resilience and healing abilities of medicinal plants in changing environments.

#### 5.1.8. Long-Term Consequences

The investigation into the long-term repercussions of heavy metals exposure in medicinal plants aims to elucidate sustained influences on herb biology. This examination delves into the long-term impacts on the delicate equilibrium between antioxidant defenses and metal-induced stress. The focus is on the persistent alterations within the plants defense mechanisms and how prolonged exposure may affect the overall quality and therapeutic efficacy of medicinal herbs [60,88].

This examination aims to provide insights into the nuanced responses of herbs to prolonged exposure, contributing to the comprehension of the persistent consequences within the context of evolving environmental challenges. The extended exposure of medicinal plants to heavy metals can lead to cumulative effects on their antioxidant activity. With time, as the duration of exposure increases, there is a likelihood of a cumulative impact on the equilibrium between antioxidant defenses and metal-induced oxidative stress [47]. This prolonged exposure may result in a shifting balance, potentially altering the overall quality and efficacy of bioactive compounds within the medicinal plants. Understanding these cumulative effects is critical for assessing the long-term consequences of heavy metal exposure on the antioxidant capacity of medicinal plants and, consequently, their therapeutic potential [124,147].

#### 5.1.9. Interactions among Secondary Metabolites

Beyond affecting antioxidant pathways, heavy metal stress extends its impact to compromise biosynthetic pathways responsible for various secondary metabolites. Heavy metal stress prompts a strategic response that involves the modulation of biosynthetic pathways for compounds like phenolics, flavonoids, and alkaloids. These secondary metabolites, recognized for their diverse bioactive properties, undergo alterations in their synthesis due to the redirection of resources away from these pathways [67]. This interaction between pathways not only signifies a disturbance in the complex network of biochemical processes but also raises implications for the overall chemical profile of medicinal plants. So, it is important to explore the compromised biosynthetic equipment, revealing altered concentrations of secondary metabolites due to heavy metal exposure, as follows [3,67]:

*(i) Compromised biosynthetic pathways*: The impact of heavy metal stress extends beyond antioxidant pathways, affecting the biosynthesis of various secondary metabolites in medicinal plants. This interdependence between pathways signifies a disruption in the complicated network of biochemical processes [124,172]. Heavy metal stress can compromise biosynthetic pathways, potentially leading to altered concentrations of secondary metabolites [37,117] This interference in biosynthetic equipment has implications for the overall chemical profile of medicinal plants, highlighting the need to explore the broader consequences of heavy metal exposure on their secondary metabolite composition, insufficiently addressed until now [67].

*(ii) Phenolic and alkaloid synthesis variations*: Central to the pharmacological properties of medicinal plants, phenolic compounds and alkaloids may undergo variations in response to heavy metal exposure. The complex balance between antioxidant compounds and other secondary metabolites, such as phenolics and alkaloids, may be dynamically regulated under the influence of environmental stress [4,117]. Fluctuations in the concentrations of these key compounds represent a tangible manifestation of the cross-talk between pathways, revealing the interrelation of biochemical processes within medicinal plants. The involved interaction between heavy metals and medicinal plants extends to changes in gene expression and enzyme activities involved in alkaloid biosynthesis. These alterations can have discernible effects on the concentrations of alkaloids in plants, shaping their bioactive profile under the influence of heavy metal stress [166].

In essence, the dynamics of secondary metabolite pathways under heavy metal stress reflect a nuanced interaction between environmental challenges and the plant’s biochemical responses. The modulation of biosynthetic pathways for phenolics, flavonoids, and alkaloids reveals the complicated strategies employed by medicinal plants to cross the complexities posed by heavy metal exposure, providing insights into the adaptive mechanisms shaping their secondary metabolite composition [67]. Understanding these variations is essential for unraveling the comprehensive impact of heavy metal stress on the secondary metabolite composition and, consequently, the therapeutic potential of medicinal plants.

#### 5.1.10. Cellular Compartmentalization

Certain medicinal plants employ strategies addressing cellular compartmentalization in response to heavy metal stress, which serves as complex mechanisms for minimizing the direct impact of heavy metals on cellular functions [121]. An insight in this mechanism can contribute to the broader knowledge of adaptive responses in medicinal plants, elucidating the complex ways in which cellular compartments play a role in safeguarding against environmental stressors:

*(i) Vacuolar sequestration*: In response to heavy metal stress, certain medicinal plants employ cellular compartmentalization strategies, sequestering heavy metals within vacuoles. This sequestration process serves as a protective mechanism to minimize the direct impact of heavy metals on cellular functions. However, the vacuolar sequestration of heavy metals may have broader implications for the availability of metals to interact with cellular antioxidants [173,174]. This, in turn, can modulate the antioxidant response within the plant, influencing its ability to counteract metal-induced oxidative stress.

*(ii) Plastidial responses*: Cellular compartments such as plastids, including chloroplasts, emerge as sensitive targets under heavy metal stress. Alterations in plastidial functions can have cascading effects, particularly on the synthesis of crucial antioxidants like chlorophylls and carotenoids. These pigments contribute significantly to the plant’s antioxidant defense system [175]. Modulation of plastidial processes not only impacts pigment synthesis, but also influences the overall redox status of the plant [176]. Understanding the cellular compartmentalization strategies, especially in vacuoles and plastids, provides valuable insights into the adaptive responses of medicinal plants to heavy metal stress and the elaborate ways in which cellular functions are finely tuned to mitigate the impact of environmental challenges.

### 5.2. Other Potential Pathways and Interactions Involved in the Impact of Heavy Metals on Medicinal Plants’ Antioxidant Activity

#### 5.2.1. Cellular Signaling Networks

Within the domain of cellular signaling networks, a dynamic interaction unfolds as heavy metal stress engages with complex cellular processes. One noticeable aspect involves calcium signaling, where heavy metal exposure induces fluctuations in cellular calcium levels, setting off cascades of signaling events. Calcium, functioning as a secondary messenger, complexly influences various cellular responses, notably activating pathways associated with antioxidant defense. This interconnected signaling network becomes a central element in the plants adaptive mechanisms to counter the oxidative stress induced by heavy metals [177].

Concurrently, the activation of Mitogen-Activated Protein Kinases (MAPKs) emerges as a critical link in cellular signaling pathways under heavy metal stress. This activation establishes a complex interference between MAPKs and hormonal signaling pathways within the plant. The interplay between MAPKs and plant hormones, including abscisic acid (ABA), salicylic acid (SA), and ethylene (ET), orchestrates the adaptive responses to heavy metal-induced oxidative stress. This challenging coordination of signaling networks becomes a defining feature in the plants capacity to interpret and respond to the multidimensional challenges posed by heavy metal exposure [178,179]. The exploration of cellular signaling networks can evidence the sophisticated ways in which plants cope with heavy metal stress. Calcium signaling and crosstalk between MAPKs and hormones activate defenses against oxidative stress caused by heavy metals.

#### 5.2.2. Metabolic Reprogramming

In the exploration of metabolic reprogramming under heavy metal stress, a nuanced adjustment occurs within the complicated mechanism of plant metabolism. This phenomenon is particularly evident in the modulation of carbon and nitrogen metabolism, where heavy metal exposure activates a reprogramming process [180,181]. The allocation of resources within these metabolic pathways becomes a focal point, complexly influencing the availability of precursors essential for the synthesis of antioxidant compounds. This reprogramming thus becomes a key aspect of the plants adaptive strategy, allowing it to tailor its metabolic responses to the specific challenges posed by heavy metal stress.

Simultaneously, the tricarboxylic acid (TCA) cycle assumes a central role in the orchestration of metabolic responses, especially in the context of redox regulation [182,183]. Heavy metals exert an influence on the TCA cycle, potentially impacting the availability of reducing equivalents. This alteration within the TCA cycle resonates in the broader redox status of the plant, complexly modulating its antioxidant responses. The relationship between metabolic reprogramming and redox regulation becomes a defining feature, exemplifying the plants ability to recalibrate its metabolic mechanism to counteract the oxidative stress induced by heavy metals [86,162].

In essence, the examination of metabolic reprogramming offers insights into the adaptive strategies employed by plants under heavy metal stress. The adjustments within carbon and nitrogen metabolism, coupled with the influence on the TCA cycle, jointly underline the plants capacity to dynamically allocate resources and modulate its metabolic pathways. This reprogramming phenomenon becomes a key component in the complicated web of responses arranged by plants to cross the multifaceted challenges posed by heavy metal exposure.

#### 5.2.3. Redox-Sensitive Transcription Factors

In the field of redox-sensitive transcription factors, a distinct layer of regulation emerges as plants respond to the challenges of heavy metal stress. One key player in this regulatory network is the Nuclear Factor-E2-Related Factor (Nrf2), recognized for its redox sensitivity. Nrf2 assumes a key role in coordinating the cellular defense against heavy metal-induced oxidative stress. As heavy metals impose their impact, oxidative stress ensues, activating Nrf2 [184,185]. This activation initiates a cascade of events, leading to the transcription of genes responsible for antioxidant and detoxification processes. Nrf2, as a redox-sensitive transcription factor, becomes a central architect in the plants adaptive strategies, finely tuning gene expression to counteract the reactive species generated under heavy metal exposure [186,187].

Concurrently, the APETALA2/Ethylene-Responsive Factor (AP2/ERF) transcription factors step into the spotlight within the intricate network of stress responses, including those induced by heavy metals [188,189]. These factors, essential components in the plants regulatory machinery, govern the expression of genes associated with antioxidant defense. Their role extends to balancing the cellular redox status, ensuring a harmonious equilibrium within the plants physiological responses to heavy metal stress. The AP2/ERF transcription factors, in their redox-sensitive modulation, exemplify the complex regulatory background through which plants cope with the challenges posed by heavy metal exposure [190,191].

#### 5.2.4. Interactive Root-Shoot Signaling

Concerning the interactive root-shoot signaling, a sophisticated system develops as plants cross the challenges posed by heavy metal stress. Root cells, when exposed to heavy metals, develop a strategic response by releasing signaling molecules. These molecular messengers traverse upward, reaching aerial parts of the plant and thereby influencing systemic responses. This root-to-shoot signaling, a fundamental communication channel, assumes a focal role in coordinating antioxidant defenses across the entire plant. Simultaneously, it optimizes resource allocation, ensuring an adaptive response that encompasses the entirety of the plant in the face of heavy metal-induced stress [155,192].

Within this complicated signaling network, phloem-mediated signaling emerges as a critical player. The phloem, responsible for transporting signaling molecules and nutrients between different plant organs, becomes a channel for the systemic communication of stress signals induced by heavy metals. The stress imposed by heavy metals can modulate phloem-mediated signaling, exerting an influence on the systemic distribution of antioxidants and their precursors. This modulation adds another layer of complexity to the plant’s systemic response, as it strategically adjusts the distribution of resources to counteract oxidative stress throughout the plant [193,194,195].

The root-to-shoot signaling pathway, coupled with the phloem-mediated communication network, collectively exemplifies the plant’s ability to integrate responses across its entire structure. This integration, coordinated through complex signaling pathways, becomes a basis in the plants adaptive strategies to harmonize antioxidant defenses and resource allocation in the presence of heavy metal-induced challenges.

#### 5.2.5. Post-Transcriptional Regulation

In the multifaceted context of post-transcriptional regulation, plants deploy a refined regulatory network to fine-tune gene expression under the influence of heavy metal stress. MicroRNAs (miRNAs), small RNA molecules with potent regulatory roles, emerge as key players in this regulatory ensemble. Under heavy metal stress, alterations in the expression of miRNAs become apparent, exerting influence on the abundance of target transcripts [161,196]. Notably, these transcripts are integral components of antioxidant pathways and secondary metabolism. The post-transcriptional modulation mediated by miRNAs becomes a dynamic element in the plant’s response, allowing it to delicately adjust gene expression to counteract the oxidative stress induced by heavy metals [197].

Concomitantly, RNA-binding proteins step into the focus as essential contributors to post-transcriptional gene regulation.

Under heavy metal stress, changes in the activity of these proteins come to the fore, impacting mRNA stability and translation efficiency. These nuanced adjustments, orchestrated by RNA-binding proteins, contribute significantly to the fine-tuning of antioxidant responses within the plant [198]. The post-transcriptional regulation mediated by these proteins acts as a precision tool, allowing the plant to dynamically adapt its gene expression profile to the specific challenges posed by heavy metal-induced stress.

#### 5.2.6. Synthetic Biology Approaches

Within the domain of synthetic biology approaches, cutting-edge methodologies empower researchers to engineer novel strategies for optimizing plant responses, especially in the context of heavy metal stress. Metabolic engineering, a cornerstone of synthetic biology, emerges as a potent tool for elevating antioxidant production in medicinal plants. Through the judicious application of genetic engineering techniques, metabolic pathways can be tailored to enhance the plants inherent antioxidant defenses. This approach not only fortifies the plant’s ability to counter oxidative stress, but also ensures a robust antioxidant response, even in the presence of heavy metals [199,200].

A revolutionary tool in the synthetic biology toolkit is the CRISPR/Cas9 technology, offering precise and targeted genome editing capabilities. Leveraging this system, researchers can undertake careful modifications to specific genes linked with antioxidant pathways [201,202]. This targeted genetic editing enables the enhancement of the plants intrinsic capacity to cope with heavy metal-induced oxidative stress. By selectively manipulating genes associated with antioxidant defenses, researchers can fine-tune the plants molecular machinery, preparing the way for tailored adaptations to thrive in metal-contaminated environments [119,203].

Consequently, synthetic biology approaches represent a frontier in advancing the resilience of medicinal plants against the challenges posed by heavy metal stress. Metabolic engineering and CRISPR/Cas9 technologies offer unprecedented precision in enhancing antioxidant responses, providing innovative solutions for ensuring the sustainability and therapeutic potential of medicinal plants in environments affected by heavy metal contamination.

#### 5.2.7. Ecosystem-Level Dynamics

In the context of ecosystem-level dynamics, medicinal plants can play a key role, especially when equiped with robust antioxidant systems. The phytoremediation potential of these plants comes to the forefront, showcasing their ability to sequester heavy metals and alleviate oxidative stress. This capability contributes significantly to ecosystem-level dynamics, exerting influence on soil health and the availability of nutrients. Medicinal plants, with enhanced antioxidant systems, become pivotal players in shaping the ecological landscape by actively participating in the remediation of heavy metal-contaminated environments [204,205].

Beyond their direct interactions with heavy metals, medicinal plants engage in complex biotic relationships that further amplify ecosystem-level dynamics. Biotic factors, ranging from herbivores to symbiotic relationships, become integral components of the plants response to heavy metal stress. The impact of heavy metals extends beyond the plant itself, influencing the expression of antioxidant defenses and the synthesis of bioactive compounds. This interaction with other organisms adds layers of complexity to the ecosystem, where the responses of medicinal plants to heavy metals reverberate through biotic interactions, potentially shaping the overall dynamics of the ecosystem [206].

In essence, examining ecosystem-level dynamics reveals the multifaceted contributions of medicinal plants to environmental resilience. Their phytoremediation potential and complex biotic interactions underscore the broader implications of their responses to heavy metal stress. Understanding these dynamics provides insights into the interconnected relationships between medicinal plants, heavy metals, and the broader ecosystem, offering a holistic perspective on the role of these plants in maintaining ecological balance in environments affected by heavy metal contamination.

## 6. Implications of Heavy Metal-Induced Alterations on Bioactive Compounds and Therapeutic Potential of Medicinal Plants

### 6.1. Effects of Heavy Metals Stress on Bioactive Compounds Changes and Their Therapeutic Capacity

The implications of heavy metal-induced changes on the overall quality and efficacy of medicinal plants constitute a critical area of study with far-reaching consequences for human health and environmental well-being. Heavy metals, pervasive in various ecosystems due to anthropogenic activities, can profoundly impact the physiological and biochemical attributes of medicinal plants. As these plants serve as reservoirs for bioactive compounds with therapeutic potential, understanding the consequences of heavy metal exposure is paramount.

Heavy metal-induced alterations encompass diverse facets, ranging from changes in antioxidant defenses and secondary metabolite pathways to modifications in gene expression and metabolic reprogramming. The problem is initiated at the roots, where medicinal plants absorb heavy metals from the soil, starting a cascade of events that influence metal uptake, translocation, and subsequent responses at the cellular and molecular levels. The complicated relationship between heavy metals and medicinal plants reveals a complex situation, shaping the synthesis of bioactive compounds, antioxidant defenses, and overall plant health, as illustrated in Table 3.

This exploration addresses the nuanced responses of medicinal plants to heavy metal stress, explaining how these changes resonate through the plants biochemistry and physiology. As heavy metals can disrupt key pathways involved in the synthesis of therapeutic compounds, there is a potential impact on the overall quality and efficacy of medicinal plants. Unraveling these implications is not only vital for understanding the involved mechanisms at play but also for developing strategies to mitigate the adverse effects and ensure the sustainability of medicinal plant resources in the face of environmental challenges. For example, [77] analyzed the various effects of Cd, one of the most toxic heavy metal, on some medicinal plants growth, and found that the presence of Cd in various concentration can affect germination, vegetative and reproductive growth (Table 4).

The implications of heavy metal-induced changes on medicinal plants extend across ecological, socio-economic, and regulatory dimensions. Addressing these implications requires a comprehensive and interdisciplinary approach that involves researchers, communities, industry stakeholders, and policymakers. As we traverse the complex relationship between environmental health and medicinal plant quality, a commitment to sustainable practices, innovative technologies, and global cooperation is essential to safeguard both human well-being and the integrity of traditional medicine.

Therefore, heavy metal-induced changes in medicinal plants can have profound implications for their overall quality and efficacy. Balancing the need for environmental conservation, sustainable plant use, and ensuring the safety and efficacy of herbal medicines requires a multidisciplinary approach. Ongoing research is crucial for understanding the complex interactions between heavy metals and medicinal plants and developing strategies to mitigate the adverse effects on their therapeutic potential.

### 6.2. Potential Risks for Human Consumption of Medicinal Plants Contaminated with Heavy Metals

**Figure 5:**
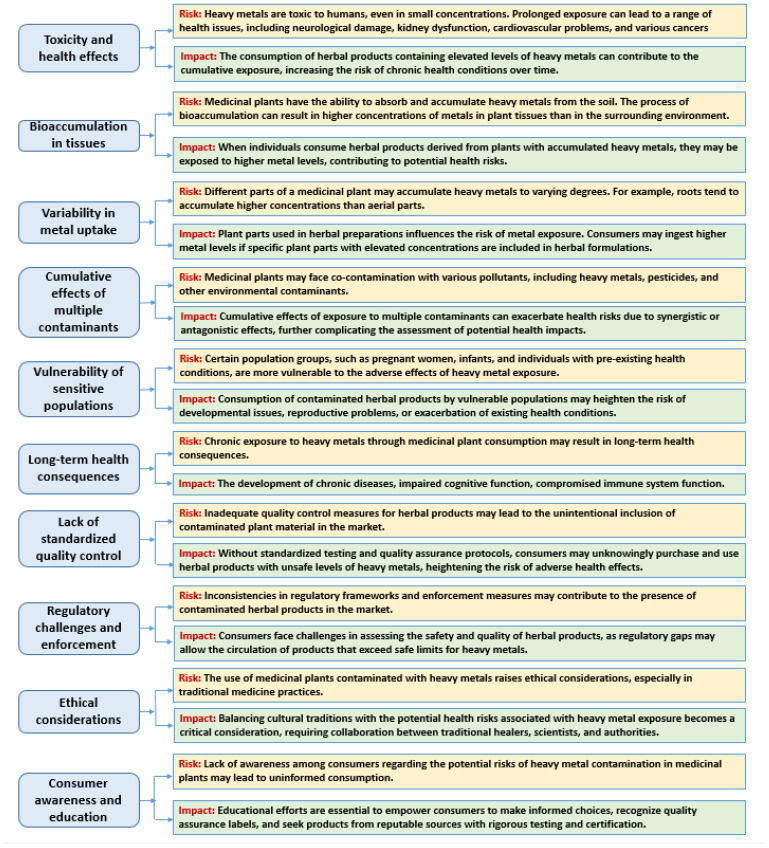
Potential risks associated with the consumption of medicinal plants contaminated with heavy metals.

The contamination of medicinal plants with heavy metals poses significant risks for human consumption. Heavy metals, even in trace amounts, can accumulate in plant tissues and make their way into herbal preparations, potentially causing adverse health effects, generating potential risks associated with the consumption of medicinal plants contaminated with heavy metals, emphasizing the need for a comprehensive understanding of associated challenges (Figure 5).

Heavy metals, including lead, mercury, cadmium, and arsenic, exhibit toxicity even at low concentrations, contributing to a range of health issues such as neurological damage, kidney dysfunction, cardiovascular problems, and various cancers. Prolonged exposure, particularly through herbal products, can lead to cumulative health risks over time. Medicinal plants possess the capacity to absorb and accumulate heavy metals, resulting in higher concentrations within plant tissues than in the surrounding environment [81,132]. The consumption of herbal products derived from these plants may unknowingly expose individuals to elevated metal levels, contributing to potential health risks. Different parts of a medicinal plant may accumulate heavy metals to varying degrees, with roots often having higher concentrations than aerial parts. The choice of plant parts in herbal formulations influences the risk of heavy metal exposure, impacting consumers who may unknowingly ingest higher metal levels [54,93].

Medicinal plants may face co-contamination with various pollutants, complicating the assessment of potential health impacts. The cumulative effects of exposure to multiple contaminants, including heavy metals and pesticides, can exacerbate health risks through synergistic or antagonistic interactions. Certain population groups, with pre-existing health conditions, are more vulnerable to the adverse effects of heavy metal exposure. Consumption of contaminated herbal products by these populations may heighten the risk of developmental issues, reproductive problems, or exacerbation of existing health conditions [68].

Chronic exposure to heavy metals through medicinal plant consumption may lead to long-term health consequences, including the development of chronic diseases, impaired cognitive function, and compromised immune system function. Inadequate quality control measures for herbal products may result in the unintentional inclusion of contaminated plant material in the market (Hlihor et al., 2022; Asiminicesei et al., 2020) [78,81]. Without standardized testing and quality assurance protocols, consumers may unknowingly purchase and use herbal products with unsafe levels of heavy metals. Inconsistencies in regulatory frameworks and enforcement measures may contribute to the presence of contaminated herbal products in the market. Consumers face challenges in assessing the safety and quality of herbal products, as regulatory gaps may allow the circulation of products that exceed safe limits for heavy metals [99].

The use of medicinal plants contaminated with heavy metals raises ethical considerations, especially in traditional medicine practices. Balancing cultural traditions with potential health risks necessitates collaboration between traditional healers, scientists, and regulatory authorities. Lack of awareness among consumers regarding the potential risks of heavy metal contamination in medicinal plants may lead to uninformed consumption. Educational efforts are essential to empower consumers to make informed choices, recognize quality assurance labels, and seek products from reputable sources with rigorous testing and certification.

## 7. Methodology

This section outlines the information sources used, the electronic search strategy, the study selection process, methodologies to minimize bias, data extraction methods, and additional analyses conducted [207]. By providing this information, the methodology section ensures transparency and replicability of the study. It allows readers to understand how the research was conducted, the criteria used to select relevant studies, and the steps taken to minimize bias. This section also highlights the rigor and scientific integrity of the study, as well as the comprehensive approach taken to gather and analyze data. Overall, the methodology section is essential in establishing the credibility and validity of the research findings.

### 7.1. Information Sources

In this study, a comprehensive search was conducted to identify relevant information sources. The following information sources were utilized:

- Databases: Major databases such as ScienceDirect, Springer, Wiley, PubMed, Scopus, and Web of Science were searched to retrieve relevant studies. These databases cover the period from 2010 to 2024.
- Date last searched: The databases were last searched in 2015 to ensure that the most up-to-date studies were included in the review.

### 7.2. Electronic Search Strategy

The full electronic search strategy for at least one major database, such as ScienceDirect, is presented below [208]:

- Search terms: antioxidant defense mechanisms; bioactive compound synthesis; environmental factors; health risks; epigenetic modifications; oxidative stress; redox equilibrium; regulatory challenges; root-shoot signaling
- Limits: publication date: 2010–2024, language: English
- Boolean operators: the use of AND, OR, NOT operators
- Search syntax: e.g., Web of Science: TS = (“medicinal plants” AND “antioxidants” AND “heavy metal”) AND PY = 2010–2024; e.g., Scopus: TITLE-ABS-KEY(“medicinal plants” AND “antioxidants” AND “heavy metal”) AND PUBYEAR > 2010 AND PUBYEAR < 2024; ScienceDirect: e.g., TITLE-ABS-KEY(“medicinal plants” AND “antioxidants” AND “heavy metal”) AND PUBYEAR > 2010 AND PUBYEAR < 2024

The search strategy was designed to be comprehensive and specific, aiming to capture all relevant studies related to the topic of heavy metal toxicity and antioxidant content in medicinal plants. The strategy was documented in detail so that it could be repeated by other researchers.

### 7.3. Study Selection Process

The process for selecting studies involved several stages to ensure the inclusion of relevant and high-quality studies. The selection process included the following steps:

(i) Initially, all identified studies were screened based on their titles and abstracts to determine their relevance to the research question. Irrelevant studies were excluded at this stage.

(ii) The remaining studies underwent a full-text review to assess their eligibility for inclusion in the systematic review. Studies that met the predefined inclusion criteria were selected for further analysis.

(iii) The selected studies were included in the systematic review, where their findings and methodologies were critically evaluated and synthesized.

The selection process was conducted independently by three reviewers to minimize bias and ensure consistency. Any discrepancies or disagreements were resolved through discussion and consensus.

### 7.4. Methodologies to Minimize Bias

The methodologies used in this review were designed to minimize bias and ensure the use of research with a minimal degree of bias. The approaches which were employed are described below.

#### 7.4.1. Comprehensive Search

The comprehensive search conducted in this study aimed to identify all relevant studies on the complex relationship between heavy metal toxicity and the antioxidant content in medicinal plants. The researchers developed and implemented a robust search strategy to minimize the risk of publication bias and ensure a comprehensive review of the available literature. The search strategy involved searching major databases such as ScienceDirect, Scopus, Web of Science etc. These databases were selected for their extensive coverage of scientific literature. The search terms used were carefully chosen to capture relevant studies, and Boolean operators (such as AND, OR, NOT) were used to combine and refine the search terms. The search strategy also included specific limits, such as publication date and language, to focus on recent studies and those published in English. This helped ensure that the most up-to-date and accessible research was included in the review. The databases were last searched on a specific date to ensure that the most recent studies were included in the review. This helped ensure that the findings were based on the most current and relevant evidence available.

#### 7.4.2. Inclusion Criteria

Inclusion criteria are specific criteria or characteristics that studies must meet in order to be included in the review. Clear and predefined inclusion criteria were established to guide the selection of studies, minimizing the potential for bias in study selection. These criteria were established in advance to ensure that the selection process is transparent, systematic, and unbiased, to minimize the risk of selecting studies that may introduce bias or skew the results. The inclusion criteria were carefully determined in advance to ensure that the selection process was transparent, systematic, and unbiased.

The specific inclusion criteria used in this study included factors such as study design, population characteristics, intervention or exposure of interest, outcome measures, and language or publication status. These criteria were selected based on the research question and objectives of the study. For example, the study included both theoretical and experimental studies that investigated the relationship between heavy metal toxicity and the antioxidant content in medicinal plants. The intervention or exposure of interest have focused on the effects of heavy metal exposure on plant growth or antioxidant production. The outcome measures included biochemical or physiological parameters related to heavy metal toxicity and antioxidant content. Additionally, the inclusion criteria specified that only studies published in English were considered, and also those studies published within a certain time frame were included (the last 10 years, prevalently). These criteria were established to ensure that the selected studies were relevant and appropriate for the review.

#### 7.4.3. Risk of Bias Analysis

The purpose of this analysis was to ensure that the findings of the studies are reliable and unbiased. The risk of bias analysis involved critically evaluating the selected studies to identify any potential sources of bias that could affect the validity and generalizability of the findings. A systematic approach to evaluate the potential biases was taking the following steps:

(i) Definition of the criteria for analysis: the authors established clear criteria for assessing the risk of bias, which included: study design, sample size, data collection methods, data analysis, and reporting of results.

(ii) Review study characteristics: the authors carefully examined the characteristics of each study, such as the study design, population, intervention, and outcome measures to understand the potential sources of bias that may be present in the study.

(iii) Evaluation of study quality: the authors critically evaluated the quality of each study, assessing whether the study design and methods are appropriate for addressing the research question and whether the data collection and analysis are conducted rigorously in the literature surveyed.

(iv) Identification of potential biases: the authors identified any potential biases (selection bias, performance bias, detection bias, attrition bias, reporting bias) that may affect the validity and reliability of the study findings. These biases were avoided or removed to elude their impact on the internal and external validity of the study. This analysis was a qualitative only, since it didn’t used specific tools like the Cochrane Risk of Bias Tool, the Newcastle-Ottawa Scale or others.

(v) Data synthesis: The risk of bias information obtained from the assessment was considered during the data synthesis process to ensure that the findings were interpreted in light of the quality and reliability of the included studies.

### 7.5. Data Extraction

The method of data extraction involved the use of piloted forms and was independently performed by two reviewers. The data extraction process aimed to capture relevant information from the included studies, such as study characteristics, intervention details, outcome measures, and results. Any discrepancies or disagreements in data extraction were resolved through discussion and consensus.

### 7.6. Additional Analyses

The authors likely employed qualitative approaches such as literature reviews, case studies, or qualitative interviews to gather additional insights. These methods allow for a more in-depth exploration of the topic, focusing on the qualitative aspects rather than quantitative measurements.

By conducting these additional analyses, the authors aimed to gain a comprehensive understanding of the impact of heavy metal toxicity on medicinal plants. They have explored factors such as the nuanced responses of different plant species to prolonged exposure, the potential of targeted genetic editing to enhance plant resilience, and the broader consequences of heavy metal exposure on the secondary metabolite composition of medicinal plants.

The qualitative nature of these analyses allows for a more holistic understanding of the subject matter, capturing the complexities and different nuances of the study. These additional analyses focused on several key aspects:

(a) Impact on antioxidant capacity: The authors explored how heavy metal stress affects the antioxidant capacity of medicinal plants. They examined the physiological and biochemical responses of these plants to heavy metals, highlighting how metal stress disrupts biosynthetic pathways and alters the concentrations of secondary metabolites. This disruption can potentially compromise the overall quality and efficacy of medicinal plants.

(b) Biosynthetic pathways: The authors investigated the complex relationship between heavy metals and biosynthetic pathways critical to the therapeutic potential of medicinal plants. They examined how heavy metal stress can disrupt these pathways, leading to changes in the production of secondary metabolites. Understanding these disruptions is important for ensuring the usefulness of medicinal plants.

(c) Variations in plant responses: The authors examined the diverse responses of different plant species to heavy metal exposure. They documented variations in plant physiology, biochemistry, and genetics, showcasing the resilience and versatility of plant life in the face of environmental stressors. This analysis provides insights into the adaptive mechanisms of plants and helps in understanding the complex interplay between plant responses and heavy metal toxicity.

Overall, the additional qualitative analyses conducted in this document contribute to a comprehensive understanding of the topic, revealing various aspects of heavy metal stress on medicinal plants and emphasizing the importance of considering qualitative factors alongside quantitative measurements.

## 8. Conclusions

This paper provides an in-depth exploration of the complex relationship between heavy metal toxicity and the antioxidant content in medicinal plants. The research reveals that heavy metals can disrupt key pathways involved in the synthesis of therapeutic compounds, potentially affecting the quality and efficacy of medicinal plants.

The study highlights that heavy metal exposure can lead to cumulative effects on the antioxidant activity of medicinal plants. Over time, as the duration of exposure increases, there is a likelihood of a cumulative impact on the equilibrium between antioxidant defenses and metal-induced oxidative stress. This prolonged exposure may result in a shifting balance, potentially altering the overall quality and efficacy of bioactive compounds within the medicinal plants.

The research also investigates plants defense mechanisms and how prolonged exposure to heavy metals may affect the overall quality and therapeutic efficacy of medicinal herbs. It provides insights into the nuanced responses of herbs to prolonged exposure, contributing to the comprehension of the persistent consequences within the context of evolving environmental challenges.

Furthermore, the study discusses the potential of targeted genetic editing to enhance plant resilience against heavy metal stress by manipulating genes associated with antioxidant defenses. This approach represents a promising frontier in the quest to safeguard the therapeutic potential of medicinal plants in the face of heavy metal contamination. The study also underscores the importance of understanding the broader consequences of heavy metal exposure on the secondary metabolite composition of medicinal plants. Heavy metal stress prompts a strategic response that involves the modulation of biosynthetic pathways responsible for various secondary metabolites. This modulation can lead to variations in the synthesis of phenolic compounds and alkaloids, which are central to the pharmacological properties of medicinal plants.

In light of these findings, the study advocates for a comprehensive and interdisciplinary approach to address the implications of heavy metal-induced changes on medicinal plants. It emphasizes the need for robust quality control measures, regulatory frameworks, and consumer awareness to ensure the safety and efficacy of medicinal plant-based therapies. The study provides a comprehensive understanding of the complex relationship between heavy metal toxicity and the antioxidant content in medicinal plants. It underscores the need for further research and innovative approaches to safeguard the therapeutic potential of these plants in the face of environmental challenges.

## 9. Recommendations for Future Research

In order to further advance the understanding of the complex relationship between heavy metal toxicity and the antioxidant and other bioactive compound content in medicinal plants, there are several areas that could be explored in future studies.

While the existing studies have shed light on the response of certain medicinal plants to heavy metal stress, there is a need to explore a wider range of plant species. This would allow for a more comprehensive understanding of the diverse mechanisms employed by different plants to combat heavy metal toxicity and maintain their bioactive compound content.

Future studies could focus on identifying specific genes and genetic pathways that play a crucial role in the antioxidant defense mechanisms of medicinal plants. This could be achieved through techniques such as targeted genetic editing or transcriptomic analysis. Understanding the genetic basis of plant resilience to heavy metal stress would enable the development of strategies to enhance their antioxidant capacity.

Investigating the broader consequences of heavy metal exposure on the secondary metabolite composition of medicinal plants is another important area for future research. This would involve analyzing the changes in the production and accumulation of bioactive compounds, such as phenolic compounds and flavonoids, in response to heavy metal stress. Understanding these changes could provide valuable insights into the potential impact on the therapeutic properties of medicinal plants.

Future studies should also consider the broader ecological and socio-economic implications of heavy metal-induced changes in medicinal plants. This could involve assessing the effects of heavy metal contamination on plant biodiversity, ecosystem functioning, and the livelihoods of communities dependent on medicinal plants. Such studies would contribute to the development of effective conservation and management strategies.

Innovative approaches should be explored to safeguard the therapeutic potential of medicinal plants in the face of environmental challenges. This could include the development of phytoremediation techniques to mitigate heavy metal contamination in plant-growing areas, as well as the utilization of plant synthetic biology to enhance plant resilience against heavy metal stress.

By addressing these research areas, future studies can contribute to a more comprehensive understanding of the complex relationship between heavy metal toxicity and the antioxidant content in medicinal plants. This knowledge can then be applied to develop effective strategies for the conservation, management, and utilization of these valuable plant resources in the face of evolving environmental challenges.

## Figures and Tables

**Table 1:** Factors correlated with the uptake process of heavy metals by plants.

Characteristics	Factors	Description	References
**Soil characteristics**	pH levels	The pH of the soil plays an essential role in metal availability to plants. Generally, metals like lead and cadmium are more available in acidic soils, while alkaline conditions may enhance the mobility of metals like nickel and zinc. Soil pH affects metal speciation, influencing their solubility and uptake	[82,95,96]
	Soil texture	The texture of the soil, whether it’s sandy, loamy, or clayey, affects water retention and drainage. Sandy soils, for example, generally have lower cation exchange capacity (CEC) and may allow metals to leach more easily. Clay soils, with higher CEC, can retain metals, influencing their availability.	[54,89,97,98]
	Organic matter content	Soil organic matter can bind with metals, affecting their mobility and bioavailability. High organic matter content tends to reduce metal uptake by forming complexes, while low organic matter may enhance metal mobility.	[23,99,100,101]
**Metal characteristics**	Chemical form	The chemical form or speciation of metals in the soil determines their availability to plants. Metals may exist in various forms, such as free ions, complexes, or bound to soil particles. Plants tend to take up metal ions more readily than complexed or precipitated forms.	[102,103]
	Redox potential	The redox potential of the soil, indicating its oxidative or reducing conditions, affects metal speciation. For instance, in reduced conditions, metals like iron and manganese become more soluble, influencing their availability for plant uptake	[104,105]
**Plant-related factors**	Plant species	Different plant species exhibit varying affinities for specific metals. Some species are hyperaccumulators, capable of accumulating high concentrations of certain metals without significant toxicity. Understanding the metal uptake characteristics of specific plant species is critical for phytoremediation and sustainable land management.	[61,106,107]
	Root characteristics	The morphology and structure of plant roots influence metal uptake. Plants with extensive root systems can explore larger soil volumes and access metals more effectively. The presence of root filaments and mycorrhizal symbiosis can also enhance metal uptake.	[65,79,108,109,110]
	Physiological responses	Plants respond to metal stress by activating various physiological mechanisms. Metallothioneins, phytochelatins, and glutathione are examples of compounds synthesized by plants to chelate and detoxify metals, mitigating their potential harmful effects.	[83,111]
**Environmental conditions**	Temperature and moisture	Environmental conditions such as temperature and moisture influence microbial activity and the chemical reactions in the soil. These conditions, in turn, affect metal availability and uptake. Warmer temperatures may enhance microbial activity, influencing metal transformations.	[47,69,112]
	Aeration	Adequate soil aeration is essential for root respiration and microbial activity. Poor aeration, often associated with waterlogged conditions, may create reducing conditions that influence the mobility and uptake of certain metals.	[109,110]
	Competing ions	The presence of other ions in the soil solution can compete with metal ions for uptake by plant roots. High concentrations of essential nutrients or other ions may influence the bioavailability and uptake of heavy metals.	[23,73,107]

**Table 2:** Significant features involved in the diversity of plant species responses against heavy metal action.

Key Aspect	Response	Description	References
**Metal tolerance and accumulation**	Differential tolerance	Plant species vary in their tolerance to specific heavy metals. Some species demonstrate a higher tolerance to elevated metal concentrations in the soil, allowing them to thrive in metal-contaminated environments. Others may be more sensitive and susceptible to metal toxicity.	[77,87,124,125]
	Accumulation capacities	Certain plant species are adept at accumulating and sequestering heavy metals in their tissues without suffering severe toxicity. These metal-accumulating plants, known as hyperaccumulators, can store high concentrations of metals in their tissues, often as a defense mechanism against herbivores or pathogens.	[61,81,107]
**Morphological and physiological adaptations**	Root morphology	Variations in root morphology contribute to differential metal uptake among plant species. Some species may develop specialized root structures, such as root hairs or mycorrhizal associations, to enhance metal absorption and tolerance.	[79,87,126,127]
	Metal transport mechanisms	Different plant species employ distinct mechanisms for transporting heavy metals within their tissues. These mechanisms, including metal transporters and chelation processes, contribute to the variations observed in metal accumulation and distribution.	[60,66,121]
**Biochemical responses**	Metal chelation	Plants utilize various biochemical strategies to cope with metal stress. Chelation involves the binding of metals to organic molecules, reducing their toxicity. Some species may exhibit a higher capacity for metal chelation, leading to lower levels of free, toxic metal ions in plant tissues.	[50,77,111,121]
	Synthesis of metal-binding proteins	Differences in the synthesis of metal-binding proteins, such as metallothioneins and phytochelatins, contribute to species-specific responses. These proteins play critical roles in detoxifying metals and regulating metal homeostasis.	[83,111,128,129]
**Genetic variation**	Genetic diversity	Genetic factors significantly influence the responses of plant species to heavy metals. Variations in gene expression, genetic traits related to metal tolerance, and the presence of specific alleles contribute to the observed diversity in responses among different species.	[49,125,127]
	Adaptation and evolution	Over time, plant species may evolve specific adaptations to metal-contaminated environments. Natural selection favors individuals with genetic traits that confer advantages in metal-rich soils, leading to the development of metal-tolerant populations.	[81,130,131]
**Impact on growth and reproduction**	Growth inhibition	The impact of heavy metals on plant growth varies among species. Some plants may exhibit minimal growth inhibition, while others experience stunted growth, chlorosis, and other morphological abnormalities in the presence of elevated metal concentrations.	[48,59,73]
	Reproductive consequences	Heavy metal exposure can affect reproductive processes differently across plant species. Some may maintain reproductive success, while others may experience reduced seed production, impaired germination, or altered reproductive structures	[46,49,122,132]
**Soil-metal interactions**	Rhizospheric influences	The rhizosphere, the soil region influenced by root activity, plays a decisive role in mediating plant-metal interactions. Variations in root exudates and microbial communities within the rhizosphere contribute to species-specific responses to heavy metal exposure.	[69,81,133,134]
	Soil pH and composition	Soil properties, including pH and composition, interact with metal availability. Plant species may exhibit differential responses based on their preferences for specific soil conditions, influencing metal bioavailability and uptake.	[59,92,112]

**Table 3:** Consequences of heavy metal-induced modifications on the quality and effectiveness of medicinal plants.

Heavy Metal-Induced Changes	Implications	Effect
**Alterations in bioactive compound profiles**	Heavy metal stress can lead to changes in the biosynthesis of bioactive compounds, including polyphenols, alkaloids, and flavonoids, which contribute to the medicinal properties of plants	The altered profiles may result in variations in the concentration and diversity of bioactive compounds, influencing the plant’s overall pharmacological efficacy.
**Reduction in antioxidant capacity**	Heavy metals induce oxidative stress, diverting resources toward antioxidant defenses. This may lead to a reduction in the antioxidant capacity of medicinal plants.	The diminished antioxidant activity may compromise the plant’s ability to neutralize free radicals, potentially reducing its effectiveness in combating oxidative stress-related health issues.
**Changes in secondary metabolite pathways**	Heavy metal-induced disruptions in metabolic pathways can affect the synthesis of secondary metabolites, such as terpenoids and phenolics	Variations in these pathways may result in the loss or alteration of specific secondary metabolites, impacting the plant’s holistic therapeutic properties.
**Risk of contaminant accumulation**	Medicinal plants may accumulate heavy metals, especially in the roots, leading to the risk of contamination	The presence of contaminants in medicinal plant tissues may pose health risks to consumers, as the plants intended for therapeutic use may inadvertently introduce heavy metals into herbal preparations.
**Influence on medicinal plant adaptations**	Some medicinal plants may adapt to heavy metal stress by altering their physiological and biochemical responses.	While adaptations may enhance the plant’s survival in contaminated environments, they can also result in variations in the composition of bioactive compounds, affecting the plant’s traditional uses and applications.
**Compromised reproductive success**	Heavy metal stress can affect reproductive processes, leading to compromised seed production and germination.	Reduced reproductive success may impact the availability and sustainability of medicinal plant populations, affecting the long-term supply of plant material for traditional medicine and herbal industries.
**Long-term effects on plant health**	Prolonged exposure to heavy metals may exert cumulative effects on the overall health and vitality of medicinal plants	Chronically stressed plants may exhibit weakened immune responses, stunted growth, and increased susceptibility to pests and diseases, further compromising their quality and efficacy.
**Genetic and phenotypic variability**	Genetic diversity among medicinal plant populations influences their responses to heavy metals	Variability in genetic and phenotypic traits can lead to differences in the susceptibility of plant populations to heavy metal stress, resulting in variable impacts on the quality and efficacy of medicinal plants.
**Environmental considerations**	Medicinal plants grown in polluted environments may serve as phytoremediators, but their efficacy as therapeutic agents may be compromised.	The dual role of medicinal plants in environmental cleanup and traditional medicine raises ethical considerations, as the plants may accumulate contaminants while providing medicinal benefits.
**Consumer health risks**	Contaminated medicinal plants pose potential health risks to consumers due to the presence of heavy metals.	Ingesting herbal preparations containing elevated levels of heavy metals may lead to adverse health effects, counteracting the intended therapeutic benefits and undermining the safety of traditional medicine.
**Synergistic interactions with co-contaminants**	Medicinal plants often face co-contamination with multiple pollutants, including heavy metals and other environmental contaminants.	The synergistic or antagonistic interactions between co-contaminants may amplify or mitigate the impacts on the plant’s physiology, altering its biochemical composition and potentially affecting therapeutic efficacy.
**Impact on traditional knowledge and uses**	Heavy metal contamination may challenge traditional knowledge regarding the selection and use of medicinal plants.	Traditional uses based on generations of empirical knowledge may need to be re-evaluated, considering the potential variations in bioactive compound profiles and the safety of using contaminated plants for therapeutic purposes.
**Phytoremediation potential vs. medicinal quality**	Some medicinal plants are known for their phytoremediation potential, contributing to environmental cleanup	The trade-off between using plants for phytoremediation and maintaining their medicinal quality requires careful consideration. Strategies need to be developed to balance environmental benefits with the preservation of therapeutic efficacy.
**Influence on herbal industry and trade**	Heavy metal contamination can impact the herbal industry and international trade of medicinal plants.	Stringent quality control measures may be necessary to ensure that herbal products meet safety standards. The reputation of medicinal plants from specific regions may be affected if contamination issues arise.
**Pharmacological variability within plant populations**	Genetic and environmental factors contribute to pharmacological variability within medicinal plant populations.	Variability may extend to the concentration of bioactive compounds, making standardized formulations challenging. Identifying and selecting plant sources with minimal contamination becomes critical for ensuring consistent therapeutic effects.
**Regulatory considerations and quality standards**	Regulatory bodies need to adapt to evolving challenges related to heavy metal contamination in medicinal plants.	Establishing and enforcing stringent quality standards, including maximum allowable limits for heavy metals, becomes crucial to safeguarding consumer health and maintaining the integrity of herbal products in the market.
**Community health and environmental justice**	Communities relying on traditional medicine may be disproportionately affected by heavy metal contamination in medicinal plants.	Environmental justice considerations come into play, highlighting the need for sustainable land management practices, community awareness, and collaborative efforts to address both environmental and health concerns.
**Emerging technologies for monitoring and remediation**	Advancements in technology offer new tools for monitoring heavy metal contamination and remediating polluted environments.	Integrating these technologies into herbal cultivation practices can enhance the safety and quality of medicinal plants. Nanotechnology, for example, shows promise in developing efficient and eco-friendly remediation strategies.
**Community-based conservation initiatives**	Local communities are often the stewards of medicinal plant knowledge and resources.	Implementing community-based conservation initiatives that focus on sustainable cultivation practices, soil health improvement, and education can contribute to preserving the medicinal quality of plants while addressing environmental concerns.
**Global collaboration and research priorities**	Heavy metal contamination is a global issue with far-reaching implications	Prioritizing collaborative research efforts, sharing knowledge, and implementing standardized methods for assessing heavy metal levels in medicinal plants can contribute to a global understanding and management of this complex challenge.

**Table 4:** Effect of cadmium on germination, vegetative and reproductive growth in medicinal plants (Al-Khayri et al., 2023) [77].

Plant Name	Metal Concentration	Effect on Germination	Effect on Vegetative Growth	Effect on Reproductive Growth
*Adhatoda vasica* L.	0, 100, 200, 300, 400, 500, 600 ppm	na	Increasing Cd conc. had inhibitory effect on elongation, fresh and dry weight of root and shoot RRG value, leaf number, fresh weight, and area	Number, dry weight, fresh weight of inflorescence, flower bud, fruit reduced
*Alternanthera tanella* Colla	0, 50, 100, 150 mM	na	The shoots and roots reduced with increasing concentration	na
*Amaranthus spinosus* L.	5–50 ppm for 60 days	na	Significant reduction in root and shoot length and fresh weight in dose dependent manner	na
*Andrographis paniculata* (Burm.f.) Nees	10, 50, 100, 150 and 200 ppm	na	Root and stem elongation, RRG values, leaf number, dry and fresh weight of root, stem, and leaf was gradually lowered and percent phytotoxicity values increased with increasing in metal concentration	Inflorescence branch number pollen tube growth and pollen germination, flower, flower bud and fruit number n fresh weight of inflorescence and flower bud decreased
*Anethum graveolens* L.	0, 100 and 200 mM	na	Root length, leaf area, shoot and root dry weight decreased	na
*Bacopa monnieri* L.	5, 10, 50, 100 mM	na	Browning and stunting of roots with decreased biomass were observed with increasing Cd concentration	na
*Bidens pilosa* L.	2.57, 7.94, 17.33, 37.17 ppm for 40 days	na	Root and shoot biomasses gradually decreased with increasing concentration	na
*Brassica juncea* L.	200 and 300 mg L^−1^	na	Plant height, root length and biomass reduced	na
*Cannabis sativa* L.	25 mg kg^−1^ Cd for 45 days	na	Shoot and root biomass decreased with increasing concentration	na
*Catharanthus roseus* var. *rosea* L.	(0, 10, 50, 100, 200, 500 and 1000 µM	0% germination at 1000 µM concentration	The root length was inhibited	na
*Cajanus cajan* L.	1, 5, 10, 20, 50 mg L^−1^	60% reduction in seed germination with a decrease in the fresh and dry weight reduction in growth, stunting of seedlings	Reduction in fresh and dry weight and stunting of seedlings	na
*Centella asiatica* L.	50–100 ppm for 30 days	na	The root length remained the same except at 100 ppm while the shoot length increased significantly with metal concentration	na
*Cichorium pumilum* Jacq.	50, 100, 200, 400, 800, and 1600 µM	na	Hypocotyl and root length decreased with increasing Cd concentration	na
*Coriandrum sativum* L.	0, 25, 50, and 100 mg kg^−1^	Germination % (least at 50 mg^−1^ kgCd)	Root length, shoot length decreased with an elevation of Cd conc. with least at 100 mg kg^−1^ Cd	na
*Cuminum cyminum* L.	0, 300, 450, 600, 750 and 1050 µM	30% and 23% inhibition in seed germination of Isfahan and Khorasan ecotypes respectively.	43.6% and 48.7% of root growth inhibition of Isfahan and Khorasan ecotypes respectively.	na
*Drimia elata* Jacq. ex Willd.	2, 5, 10 mg L^−1^	na	The shoot and bulb dry weight reduced significantly with higher concentrations	na
*Melissa officinalis* L.	0, 10, 20 and 40 mM	na	Fresh weight increased up to 20 mM	na
*Merwilla plumbea* (Lindl.) Speta	1.5 ppm	na	The fresh weight of leaves, bulbs and roots significantly reduced	na
*Moringa oleifera* Lam.	1–5 mM for 30 days	na	The root and shoot length significantly reduced	na
*Ocimum basilicum* L.	5, 10, 15, 20, 25 ppm	na	The fresh and dry weight declined with increasing Cd concentration	na
*Ocimum basilicum* L.	0–16 mg L^−1^	4% reduction in germination at 16 mg L^−1^ Cd	na	na
*Ocimum canum* Sims.	50, 100, 150, 200, 250 mg kg^−1^	na	The root elongation and stem height inhibited	Flower number and its fresh weight and dry weight, inflorescence, fruit number. dry weight
*Phyllanthus amarus* Schumach. and Thonn.	10–100 mg kg^−1^	na	The root and shoot growth of plant remained unaffected up to 50 ppm and further decreased with the increasing concentration.	na
*Silybum marianum* L. Gaertn.	0, 100, 200, 400 and 600 mg L^−1^	14% seed germination at 600 mg L^−1^	na	na
*Trigonellafoenum-graecum* L.	0.1, 0.5, 1 and 10 mM	33% decrease in germination and no radicle growth at 10 mM	na	na
*Trigonella foenum-graecum* L.	0, 5, 15, 30, 50 mg g^−1^	na	Magnitude of increase of number of leaves, leaf area and number of branches per plant, along with shoot and root length was lowered	na
*Typha latifolia* L.	0.2–0.8 mg g^−1^	na	Leaf, shoot and root elongation and the dry weight reduced	na

(Reused upon Al-Khayri et al., (2023) [77] under the terms of the Creative Commons Attribution License (CC BY), which stipulates that the use, distribution or reproduction in other forums is permitted, provided the original author(s) and the copyright owner(s) are credited and that the original publication in this journal is cited, in accordance with accepted academic practice. No use, distribution or reproduction is permitted which does not comply with these terms).

## Data Availability

Data sharing is not applicable to this article.
